# Triphenylphosphonium derivatives disrupt metabolism and inhibit melanoma growth *in vivo* when delivered *via* a thermosensitive hydrogel

**DOI:** 10.1371/journal.pone.0244540

**Published:** 2020-12-30

**Authors:** Kyle C. Kloepping, Alora S. Kraus, Devin K. Hedlund, Colette M. Gnade, Brett A. Wagner, Michael L. McCormick, Melissa A. Fath, Dongrim Seol, Tae-Hong Lim, Garry R. Buettner, Prabhat C. Goswami, F. Christopher Pigge, Douglas R. Spitz, Michael K. Schultz

**Affiliations:** 1 Department of Radiology, Carver College of Medicine, The University of Iowa, Iowa City, IA, United States of America; 2 Department of Radiation Oncology, Free Radical Radiation Biology Program, Carver College of Medicine, The University of Iowa, Iowa City, IA, United States of America; 3 Department of Orthopedic Surgery, Carver College of Medicine, The University of Iowa, Iowa City, IA, United States of America; 4 Department of Biomedical Engineering, College of Engineering, The University of Iowa, Iowa City, IA, United States of America; 5 Department of Chemistry, The University of Iowa, Iowa City, IA, United States of America; Duke University School of Medicine, UNITED STATES

## Abstract

Despite dramatic improvements in outcomes arising from the introduction of targeted therapies and immunotherapies, metastatic melanoma is a highly resistant form of cancer with 5 year survival rates of <35%. Drug resistance is frequently reported to be associated with changes in oxidative metabolism that lead to malignancy that is non-responsive to current treatments. The current report demonstrates that triphenylphosphonium(TPP)-based lipophilic cations can be utilized to induce cytotoxicity in pre-clinical models of malignant melanoma by disrupting mitochondrial metabolism. *In vitro* experiments demonstrated that TPP-derivatives modified with aliphatic side chains accumulated in melanoma cell mitochondria; disrupted mitochondrial metabolism; led to increases in steady-state levels of reactive oxygen species; decreased total glutathione; increased the fraction of glutathione disulfide; and caused cell killing by a thiol-dependent process that could be rescued by N-acetylcysteine. Furthermore, TPP-derivative-induced melanoma toxicity was enhanced by glutathione depletion (using buthionine sulfoximine) as well as inhibition of thioredoxin reductase (using auranofin). In addition, there was a structure-activity relationship between the aliphatic side-chain length of TPP-derivatives (5–16 carbons), where longer carbon chains increased melanoma cell metabolic disruption and cell killing. *In vivo* bio-distribution experiments showed that intratumoral administration of a C^14^-TPP-derivative (12-carbon aliphatic chain), using a slow-release thermosensitive hydrogel as a delivery vehicle, localized the drug at the melanoma tumor site. There, it was observed to persist and decrease the growth rate of melanoma tumors. These results demonstrate that TPP-derivatives selectively induce thiol-dependent metabolic oxidative stress and cell killing in malignant melanoma and support the hypothesis that a hydrogel-based TPP-derivative delivery system could represent a therapeutic drug-delivery strategy for melanoma.

## Introduction

Previous reports have demonstrated that melanoma cells maintain sustained (or increased) mitochondrial oxidative phosphorylation in the presence of glycolysis [1–5]. Increased oxidative phosphorylation activity appears to result in hyperpolarization of the mitochondria membrane potential in melanoma cells relative to non-malignant cells [6,7]. Hyperpolarization of melanoma cell mitochondria has emerged as a biophysical alteration that has been suggested as a potential target for drug delivery [8,9].

**Fig 1:**
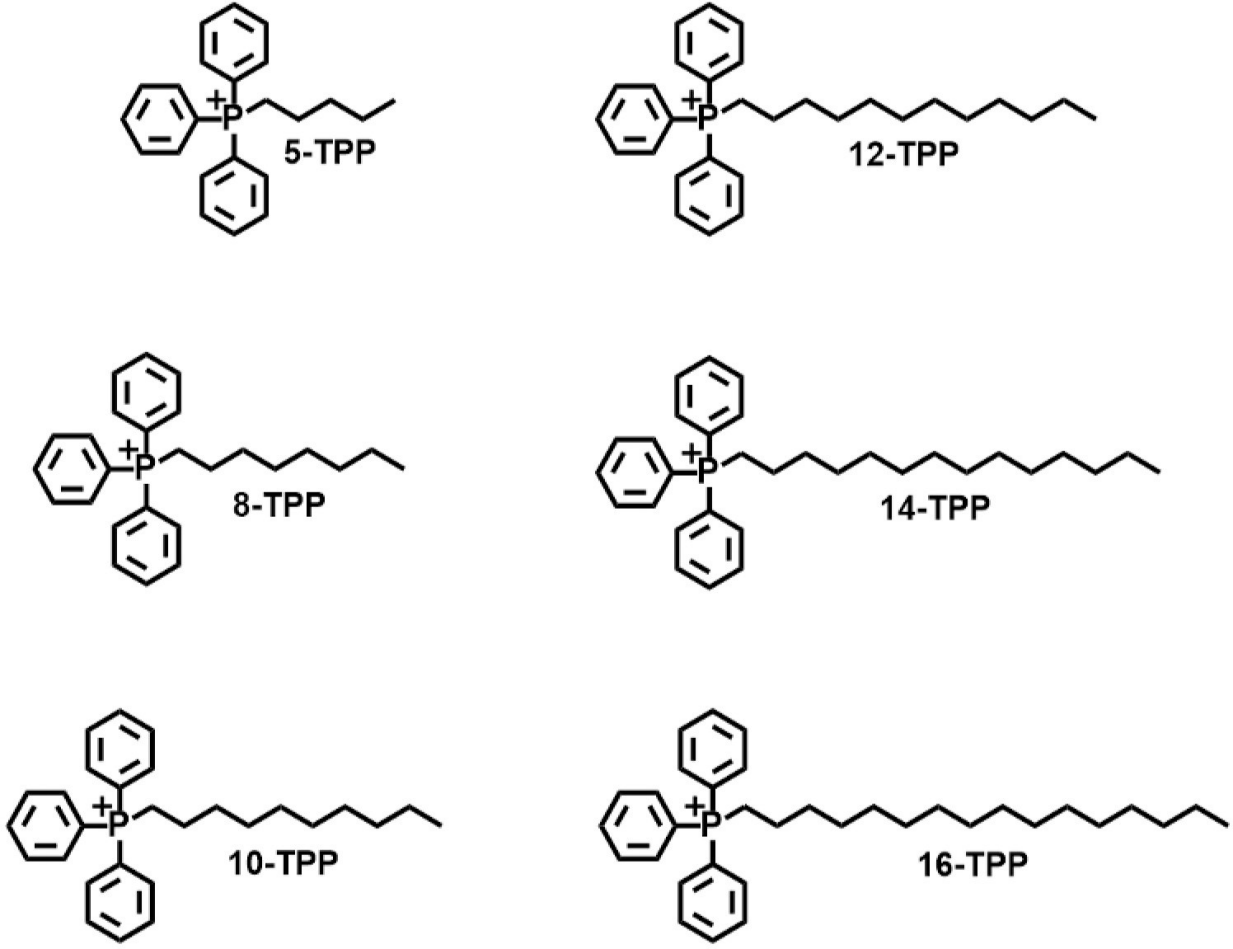
The TPP derivatives examined in this study. The positive charge of the central TPP phosphorous atom is delocalized over the lipophilic TPP moiety, contributing to the mitochondria-targeting characteristic of TPP variants. It is hypothesized that the molecular composition of the TPP side chain inserts into the mitochondrial membrane, which disrupts mitochondria metabolism.

Increased or sustained mitochondrial oxidative metabolism in melanoma cells (relative to non-malignant cells) can also result in elevated electron transport system (ETS) generated reactive oxygen species (ROS) that can lead to cytotoxic oxidative stress [10–12]. It is believed that cancer cells increase glucose uptake and glucose metabolism relative to normal cells not only to support increased energy needs, but also to detoxify chronic levels of ROS present in cancer cells [11,13,14]. Increased glycolysis results in elevated levels of glucose-6-phosphate flux through the pentose phosphate pathway in order to generate NADPH that provides the reducing equivalents for hydroperoxide metabolism via the glutathione (GSH)/glutathione peroxidase (GPx)/glutathione reductase (GR) and the thioredoxin (Trx)/peroxiredoxin (Prx)/thioredoxin reductase (TRxR) systems [10,11,13–15]. Molecular agents that are designed to exploit these metabolic differences (*e*.*g*., ETS activity, glucose metabolism, GSH, Trx) are receiving increased attention as potential interventions to target cancer cell mitochondrial redox metabolism [3,11,13,15,16]. However, because the metabolic requirements of normal cells are also potential targets, one limitation to metabolic inhibitors in cancer therapy has been systemic toxicity [15–18]. Based on these considerations, the clinical success of targeting cancer cell mitochondrial metabolism therapeutically will depend on the differential toxicity of given agents in cancer versus normal cells. Triphenylphosphonium (TPP)-based drugs have been investigated as anticancer agents due to their ability to preferentially accumulate within hyperpolarized mitochondria [19–22]. Positively charged TPP-derivatives move from the outer side of the inner mitochondrial membrane toward the mitochondrial matrix due to a positive charge delocalized over the large lipophilic TPP moiety [19–22]. Hyperpolarized membranes in melanoma cells are believed to preferentially accumulate these compounds [6,7], relative to normal cells. While several TPP-derivatives have been utilized to deliver biologically active agents to mitochondria, few studies have investigated the structure-function relationship between the length of the aliphatic side chain and cytotoxicity [4,23–25]. Furthermore, the precise mechanism of action of these compounds on mitochondrial metabolism in cancer versus normal cell cytotoxicity remains unclear [4,26]. Regardless of the TPP mechanism of action, the potential of TPP derivatives to translate clinically as cancer therapeutics depends largely on the *in vivo* pharmacodynamics and biodistribution as well as concentration achievable at the tumor site. The current study shows the differential effects of TPP derivatives of varying aliphatic chain length (from 5–16 carbons) in aggressive melanoma cells lines. These experiments showed that increasing the aliphatic chain length attached to the TPP-moiety (Fig 1) increased clonogenic cell killing proportionally with chain length up to 16 carbons. In addition, the effect of the TPP side chain length on GSSG/GSH redox couples in melanoma cells showed disruptions in the redox metabolism of thiols. Inhibition of GSH and Trx metabolism also enhanced the sensitivity of melanoma cells to TPP-derivative induced-toxicity that was reversed by the thiol antioxidant, N-acetylcysteine (NAC). Finally, the pharmacodynamics of tumor drug accumulation showed that cancer-selective growth-inhibitory levels of TPP drug accumulation *in vivo* could be most effectively achieved using a novel, locally administered, thermosensitive-hydrogel delivery system. These findings suggest that the disruption of mitochondrial metabolism using hydrogel-delivered TPP derivatives can be selectively cytotoxic to melanoma cells *in vivo* while minimizing off-target effects in non-malignant cells and highlights the potential for a TPP-hydrogel delivery system for melanoma therapy. This approach has potential in particular in the context of localized treatment of disseminated disease (*e*.*g*., brain and lymph node mets) in the adjuvant setting.

## Materials and methods

### Cell culture

A375 (CRL-1619) and MeWo (HTB-65) human melanoma cells were obtained from American Type Culture Collection (ATCC; Manassas, VA). A375 cells were cultured in DMEM (Gibco, Thermo Fisher Scientific, Waltham, MA) containing 10% fetal bovine serum (FBS; Gibco) and 1% penicillin/streptomycin (Gibco). MeWo cells were cultured in 1:1 DMEM:Ham’s F12 Nutrient Mixture (Gibco) containing 10% FBS and 1% penicillin/streptomycin (Gibco). Cells were maintained at 37°C in a humidified 5% CO_2_ incubator and detached with 0.25% trypsin-EDTA (Gibco). Experiments were performed with cells at or below passage twenty.

### Chemicals and reagents

Triphenylphosphonium compounds were obtained from Alpha Aesar (Ward Hill, MA). L-buthionine sulfoximine (BSO), auranofin (AUR), NAC, and 3-(4, 5-dimethylthiazol-2-yl)-2,5-diphenyltetrazolium bromide (MTT) were purchased from Sigma Chemical Co. (St. Louis, MO). Hydrogel was complements of Dr. Dongrim Seoul and Dr. Tae-Hong Lim at the University of Iowa. TPP and AUR were dissolved in the smallest volume of DMSO required to solubilize the compounds, while BSO was dissolved in water. NAC was dissolved in water and brought to pH 7 with sodium bicarbonate.5,6-Dichloro-2-[(*E*)-3-(5,6-dichloro-1,3-diethyl-1,3-dihydro-2*H*-benzimidazol-2-ylidene)-1-prop-1-enyl]-1,3-diethyl-1*H*-benzimidazolium iodide (JC-1) and dihydroethidium (DHE) were purchased from Molecular Probes (Eugene, OR). Replication deficient adenovirus with human copper-zinc superoxide dismutase (CuZnSOD) cDNA (Ad-CuZnSOD) driven by the cytomegalovirus (CMV) promoter was obtained from the University of Iowa DNA Vector Core (Iowa City, IA). Replication deficient adenovirus with human manganese superoxide dismutase (MnSOD), catalase (Cat), and glutathione peroxidase 4 (GPx4) cDNA (Ad-MnSOD, Ad-Cat, Ad-GPx4) driven by the CMV promoter were obtained from ViraQuest Inc. (Iowa City, IA).

### MTT cell viability assay

A375 (20,000 cells/well) and MeWo (40,000 cells/well) cells were plated in 96-well for 24 h. Cells were then treated with TPP (0.5–2.0 μM) for 24–72 h. Following treatment, medium was aspirated and MTT (5 mg/mL dissolved in 1X PBS) was added to each well and incubated for 1 h [27]. MTT was then aspirated and DMSO was added to each well to dissolve the formazan salt. The absorbance of the formazan solution was measured using a Tecan SpectraFluor Plus plate reader (Tecan, Research Triangle Park, NC) at 590 nm.

### Clonogenic cell survival assay

A375 cells were plated in 60 mm dishes at a density of 150,000 cells/dish and incubated for 48 h. Cells were then treated with 0.25–1.0 μM TPP for 24–48 h. Following drug treatment, cells were trypsinized and plated at a density of 500 cells/dish and incubated for 2 weeks. Colonies were then fixed with 70% ethanol, stained with Coomassie blue G250 (Sigma) in 45% methanol and 10% acetic acid, and counted. Only colonies ≥50 cells were counted. Surviving fraction (SF) was calculated using the following formula: SF = (number of colonies counted)/(number of cells seeded x plating efficiency).

### JC-1 mitochondria membrane potential assay

A375 cells were plated in 60 mm dishes at a density of 150,000 cells/dish and incubated for 24 h. Cells were treated with TPP (0.5–2.0 μM) for 1 h. Cells were then washed and incubated with 2.0 μM JC-1 in 1X PBS containing 5 mM pyruvate for 1 h. Control dishes were treated with 1.0 μM carbonyl cyanide 4-(trifluoromethoxy)phenylhydrazone (FCCP; Sigma) 30 min prior to measurement. Cells were washed, pelleted, and suspended in 1X PBS. Cells were analyzed using a Becton Dickinson LSR II Flow cytometer at 530 nm and 590 nm. Data from 10,000 events were collected and mean fluorescence intensity was analyzed using Flowjo software. Background fluorescence was corrected to the auto-fluorescence of unlabeled cells.

### Oxygen consumption rate measurements

A375 cells were plated in a 96-well XF96 cell culture plate at a density of 10,000 cells/well for 48 h. Medium was then replaced with Seahorse MEM medium (pH 7.4) supplemented with 25 mM glucose and 1.0 mM sodium pyruvate. Oxygen consumption rate (OCR) and extracellular acidification rate (ECAR) were measured using a Seahorse Bioscience XF96 extracellular flux analyzer (Seahorse Bioscience, Billerica, MA). Measurements were made every 10 min over the course of 1.5 h. At the 20 min mark, 1.0 μM 12-TPP was added to treatment cells only followed by the sequential addition of oligomycin (2.5 μM; Sigma) at the 55 min mark, FCCP (0.3 μM; Sigma) at the 85 min mark, and antimycin A/rotenone (5 μM; Sigma) at the 115 min mark in both control and treatment cells. Actual cell numbers in the wells was determined at the end of the experiments.

#### Enrichment of mouse liver mitochondria

Livers were harvested from mice and placed in cold homogenizing medium [0.25 M sucrose, 5 mM hepes, 0.1 mM EDTA, 0.1% fatty acid free bovine serum albumin (BSA); pH 7.25)]. Samples were homogenized on ice using a glass dounce homogenizer and centrifuged at 1000 x g for 10 min at 4°C. Supernatants were transferred to high-speed centrifuge tubes, while pellets were resuspended in cold homogenizing medium and reprocessed as described above. Supernatants were centrifuged at 10,000 x g for 10 min at 4°C. Supernatants were discarded and mitochondrial fractions were resuspended in cold potassium phosphate buffer (pH 7.25).

#### Electron transport Chain activity assay

All assays were performed as previously described at 30°C in a 1.0 mL total volume using a Beckman Coulter DU 800 Spectrophotometer (Brea, CA) [28,29]. The mitochondrial samples receiving treatment were incubated for 10 min in a high concentration of 10-TPP (500 μM to simulate the concentration of the compound in active, respirating mitochondria); however, the final 10-TPP concentration in all complex activity assays was approximately 10 μM following sample dilution after the initial incubation. Total protein content was determined by Bradford assay (Biorad) and all electron transport chain enzyme activities were normalized to the total protein content [30].

### DHE oxidation assay

A375 cells were plated in 60 mm dishes at a density of 150,000 cells/dish and incubated for 48 h. Cells were treated with 1.0 μM TPP for 1.5 h. Cells were then washed, trypsinized, and resuspended with 10 μM DHE in 1X PBS containing 5 mM pyruvate for 30 min. Control dishes were treated with 10 μM antimycin A (Sigma) 30 min prior to measurement. Samples were analyzed using a Becton Dickinson FACScan flow cytometer at 488 nm excitation and 585/42 nm emission. Data from 10,000 events were collected and mean fluorescence intensity was analyzed using Flowjo software. Background fluorescence was corrected the auto-fluorescence of unlabeled cells.

### GSH assay

A375 cells were plated in 60 mm dishes at a density of 150,000 cells/dish and incubated for 48 h. Cells were treated with 1.0 μM TPP for 24 h. Cells were then washed twice in ice-cold 1X PBS and scraped in 5% sulfosalicylic acid and stored at -80°C for future use. Samples were then thawed on ice and spun at 10,000 x g for 5 min in order to remove precipitated protein. The supernatant was collected, the volume recorded, and the pellet retained. The precipitated protein from above was resuspended in the same volume of 0.1 M sodium hydroxide (NaOH) with 1% sodium diodecyl sulfate (SDS) for future use in protein estimation and thiol levels. Total glutathione (GSH and GSSG) was measured in supernatants using a 5,5’-dithiobis-2-nitrobenzoic acid (DNTB; Sigma) spectrophotometric recycling assay as previously described [31,32]. The rate of color change was measured using a DU spectrophotometer at 412 nm for 2.5 min. Data were normalized to protein content in the resuspended pellets as determined by the bicinchoninic acid assay.

### Intracellular ATP concentration measurements

A375 cells were plated in 60 mm dishes at a density of 150,000 cells/dish and incubated for 48 h. Cells were treated with 1.0 μM TPP for 24 h. Cells were then pelleted and counted. A cell suspension containing 50,000 cells was added to the wells of a 96-well opaque wall tissue culture plate. Reagents from a CellTiterGlo ATP kit (Promega, Madison, WI) were added to lyse the cells and initiate the luminescence reaction. Luminescence was measured on a SpectraMax microplate reader. Adenosine 5’-triphosphate disodium salt hydrate (Sigma) was used to generate a standard curve of ATP concentration *vs*. luminescence signal for each assay. Intracellular ATP concentration was calculated from the corresponding standard curve run on that individual day using cell number and cell volume, as measured by cell sizing function on a Moxi Z automated cell counter (Orflow).

### NAC clonogenic cell survival assay

A375 cells were plated in 60 mm tissue culture dishes at a density of 150,000 cells/dish and incubated for 48 h. Cells were then treated with 1.0 μM TPP alone or in combination with 20 mM NAC for 24 h. Following drug treatment, a clonogenic cell survival assay was performed *vida supra*.

### GSH and TrxR inhibitor clonogenic cell survival assays

A375 cells were plated in 60 mm dishes at a density of 150,000 cells/dish and incubated for 48 h. Cells were then treated with 0.5 μM TPP alone or in combination with 100 μM BSO or 1.0 μM AUR for 24 h. Following drug treatment, a clonogenic cell survival assay was performed *vida supra*.

### Superoxide dismutase activity assay

A375 cells were plated in 60 mm dishes at a density of 150,000 cells/dish and incubated for 48 h. Cells were then transfected with Ad-CuZnSOD or Ad-MnSOD for 24 h in serum free media. The adenovirus was then removed and full media replaced for 24 h. Cells were then washed twice in ice-cold 1X PBS and scraped in 5% sulfosalicylic acid and stored at -80°C for future use. Pellets were thawed on ice and resuspended in 50 mM phosphate buffer containing 1.34 mM of the iron chelator diethylenetriaminepentaacetid acid (DETAPAC). Superoxide dismutase (SOD) activity was determined as previously described [33]. All protein levels were determined by the Lowry assay. Activity is expressed as units SOD mg protein^-1^, where one unit of SOD is the amount of protein that results in 50% of maximum inhibition.

### Catalase activity assay

A375 cells were plated in 60 mm dishes at a density of 150,000 cells/dish and incubated for 48 h. Cells were then transfected with Ad-Cat for 24 h in serum free media. The adenovirus was then removed and full media replaced for 24 h. Cells were then washed twice and scrape harvested in ice-cold 1X PBS. Pellets were thawed on ice and resuspended in 50 mM phosphate buffer containing 1.34 mM of the iron chelate DETAPAC. Cat activity was determined by measuring the rate of H_2_O_2_ removal in a DU700 spectrophotometer at 240 nm in 50 mM potassium phosphate buffer (pH 7.0). Activity is expressed as m*k* units per mg protein^-1^. Protein levels were determined by the Lowry assay.

### GPx4 activity assay

A375 cells were plated in 60 mm tissue culture dishes at a density of 150,000 cells/dish and incubated for 48 h. Cells were then transfected with Ad-GPx4 for 24 h in serum free media. The adenovirus was then removed and full media replaced for 24 h. Cells were then washed twice in ice-cold 1X PBS and scraped in 5% sulfosalicylic acid and stored at -80°C for future use. Pellets were thawed on ice and resuspended in DETAPAC. GPx4 activity was measured as previously described [34,35]. The rate of NADPH oxidation can be measured spectrophotometrically at 340 nm. One unit of GPx4 activity is defined as the amount of enzyme catalyzing 1 μM of NADPH per min and is expressed as milliunits per mg protein^-1^. Protein levels were determined by the Bradford assay.

### Adenovirus clonogenic cell survival assay

A375 cells were plated in 60 mm dishes at a density of 150,000 cells/dish and incubated for 48 h. Cells were then transfected with adenovirus (Ad-CuZnSOD, Ad-MnSOD, Ad-Cat, Ad-GPx4) for 24 h in serum free media. The adenovirus was then removed and full media replaced for 24 h. Cells were then treated with 1 μM TPP for 24 h. Following drug treatment, a clonogenic cell survival assay was performed *vida supra*.

### Tumor xenograft growth

Female 4-week-old athymic-nu/nu mice (Harlan, Indianapolis, IN) and socially housed with free access to food and water in the Animal Care Facility at the University of Iowa. The Institutional Animal Care and Use Committee at the University of Iowa approved all animal studies and procedures (ACURF # 1308161). For subcutaneous xenografts, mice were injected with 500,000 A375 cells in LEDV-free matrigel basement membrane matrix (Corning). Tumor volumes were calculated using: (length x width^2^)/2 [36]. Mice bearing tumors >15 mm in any direction or tumor ulceration were immediately euthanized via isoflurane overdose or CO_2_ exposure.

### C-14 biodistribution studies

A 12-TPP compound labeled with ^14^C at the alpha-carbon position was purchased from American Radiolabeled Chemicals (St. Louis, MO). Animals bearing A375 xenografts were administered 2.5 μCi of ^14^C labeled 12-TPP via tail vein injection, oral gavage, intraperitoneal injection, or hydrogel peritumorally. All animals were euthanized via isoflurane overdose or CO_2_ exposure at 0.5 h, 3 h, 12 h, 24 h, or 48 h post administration of ^14^C-12-TPP. Organs were harvested, dried, ground, and counted using a liquid scintillation counter (Packard). Activity was normalized to the total activity recovered in the analysis and represented as normalized activity per gram of tissue. N = 2 mice for each route of administration and time point.

### *In vivo* tumor growth studies

Athymic nu/nu mice bearing A375 melanoma xenografts (~5x5 mm in size) were administered 12-TPP suspended in hydrogel peritumorally at a 10 μM concentration. Injections were made twice weekly for three weeks. Tumor size and body weight were measured at the times of injection. Animals losing <10% of their body weight were administered 200 μL saline IP. Tumor volumes were calculated using: (length x width^2^)/2 [36]. All mice bearing tumors >15 mm in any direction or tumor ulceration were immediately euthanized via isoflurane overdose or CO_2_ exposure. N = 5 mice per group.

### TPP *in vivo* toxicity studies

Female SCID hairless mice (Harlan) were treated with 100 μM 10-TPP (or vehicle control) in their drinking water. Mice were weighed daily to monitor body weight loss to see if treatments were well tolerated. Animals losing <10% of their body weight were administered 200 μL saline IP. After 17 days, all mice were euthanized via isoflurane overdose or CO_2_ exposure and blood was drawn via a cardiac puncture and diluted with Sysmex buffer and analyzed for CBC differentials via Sysmex XT2000i Automated Hematology Analyzer per manufacturer instructions. For liver and kidney pathology, blood was immediately placed in plasma separator tubes containing heparin. After centrifugation the plasma was sent on ice to Radil Labs (Columbia, MO) for further analysis. N = 5 mice per group.

### Statistical analysis

Statistical significance for studies with more than three groups was determined using one-way ANOVA with post hoc analyses using the Tukey's honestly significant difference test for multiple comparisons. Statistical significance for studies with less than three groups was done using a Student’s t test. Homogeneity of variance was assumed at 95% confidence interval. Results with p < 0.05 were considered significant. Statistical analysis was performed using SPSS Statistics Version 21 (IBM). For *in vivo* tumor growth studies, linear mixed effects regression models were used to estimate and compare group-specific tumor growth curves. Pairwise comparisons were performed to identify specific group differences in the growth curves. All tests were two-sided and carried out at the 5% level of significance. Analyses were performed with SAS v9.4 (Cary, NC).

## Results

### Increasing TPP side-chain length decreases the viability of melanoma cells

**Fig 2:**
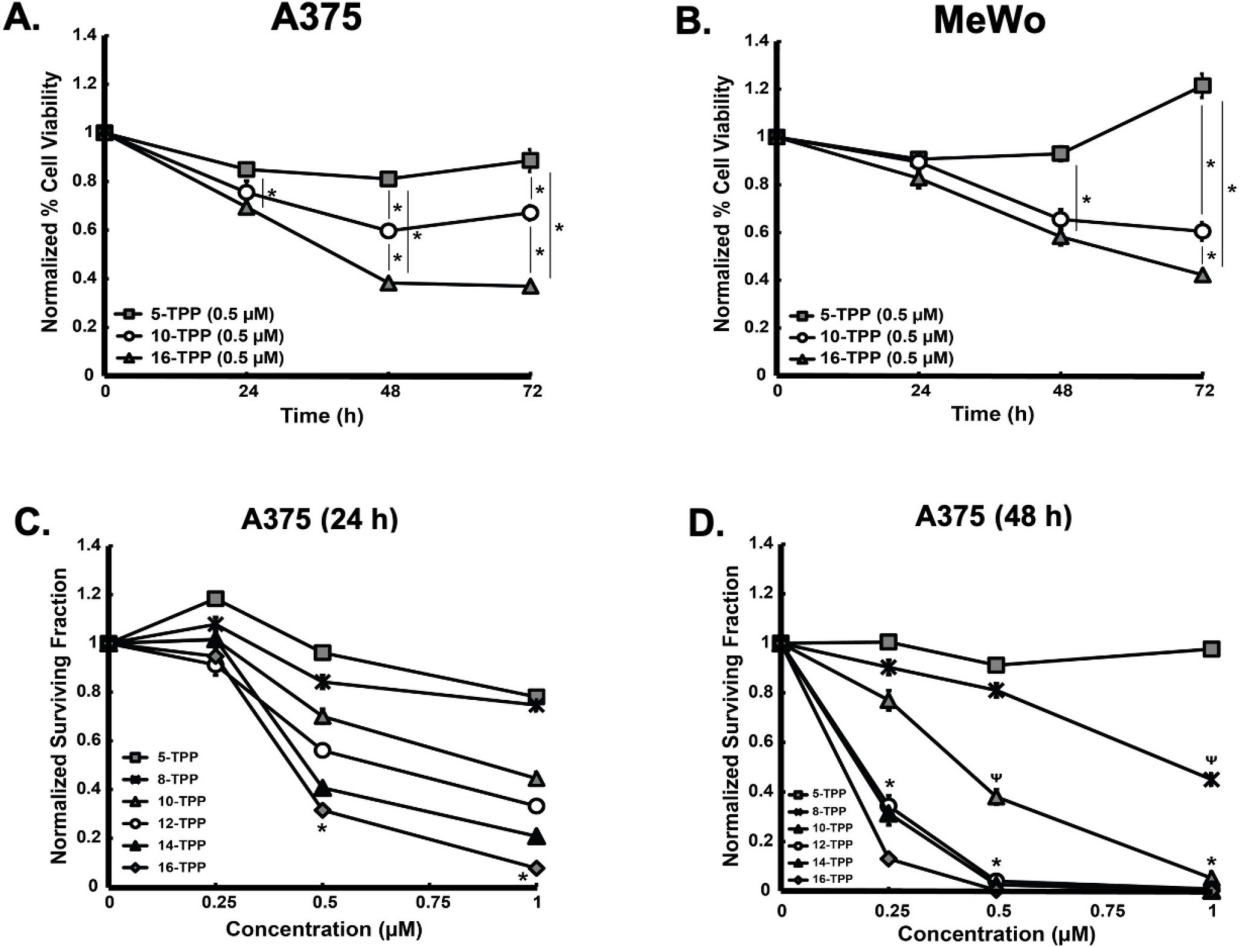
Increasing TPP side chain length decreases melanoma cell viability and clonogenic survival. (A) A375 and (B) MeWo melanoma cells were treated with 0.5 μM 5-, 8-, 10-, 12-,14-, or 16-TPP for 24 h, 48 h, or 72 h and analyzed for cell viability by the MTT method (* significant relative to control; p<0.05; N = 4). A375 melanoma cells were treated with 0.25 μM, 0.5 μM, or 1.0 μM 5-, 8-, 10-, 12-,14-, or 16-TPP for (C) 24 h and (D) 48 h and analyzed for clonogenic survival (* represents when 12-, 14-, and 16-TPP were significant relative to 5-TPP and 8-TPP; Ψ represents when 10-TPP was significant relative to 5-TPP and 8-TPP; p < 0.05; n = 3 from 2 separate experiments; N = 6). The error bars for all data presented in Fig 2 represent the mean ± SEM. Results demonstrate that TPP derivatives decrease melanoma cell viability and clonogenic survival; and there is a structure-activity relationship between TPP side chain length and decreased viability and clonogenic survival.

In order to develop an understanding of the effect of changes to TPP-aliphatic side-chain length on melanoma cell viability, MTT cell viability assays were performed in A375 (Fig 2A) and MeWo (Fig 2B) cells treated with TPP derivatives (0.5 μM) for different incubation periods. MTT analysis of the cell populations following these treatments showed a relatively modest effect on melanoma cell viability and proliferation resulting from a 24 h incubation period for all chain lengths studied. On the other hand, the viability of melanoma cells when incubated with 10-TPP and 16-TPP decreased significantly with increasing incubation periods relative to 5-TPP and control. These results support the hypothesis that lengthening of the aliphatic side chain of TPP derivatives promotes greater reductions in metastatic melanoma cell viability. It is also well established that MTT reduction is largely dependent on mitochondria NAD(P)H-dependent oxidoreductase and dehydrogenase activity of metabolically active cells [37]. Therefore, these results suggest that the decreased melanoma cell viability could be attributed to TPP targeting of melanoma cell mitochondria and subsequent decrease in mitochondrial activity.

### Increasing TPP side-chain length decreases the clonogenic survival of melanoma cells

Clonogenic survival assays were performed in addition to viability assays to determine the effect of TPP side chain length on melanoma cell reproductive integrity. For these experiments, A375 melanoma cells were treated with increasing concentrations of TPP derivatives for 24 h (Fig 2C) and 48 h (Fig 2D). Similar to MTT viability assays, clonogenic survival analysis strongly suggests that longer-length side chains impart greater reductions in melanoma cell clonogenic survival. There was no decrease in melanoma cell clonogenic survival after a 0.25 μM 24 h TPP treatment for all chain lengths studied; and the effect on melanoma cells as a result of incubation with 5-TPP was minimal at all incubation periods and concentrations employed for these experiments. On the other hand, the clonogenic survival decreased with each subsequent increase in chain length, concentration and treatment time. These results demonstrate that lengthening the aliphatic side chain of TPP derivatives results in greater toxicity as seen by clonogenic survival. Further, there is a time and concentration dependence on TPP-induced clonogenic melanoma cell death.

### Increasing TPP side-chain length decreases the mitochondrial membrane potential of melanoma cells

**Fig 3:**
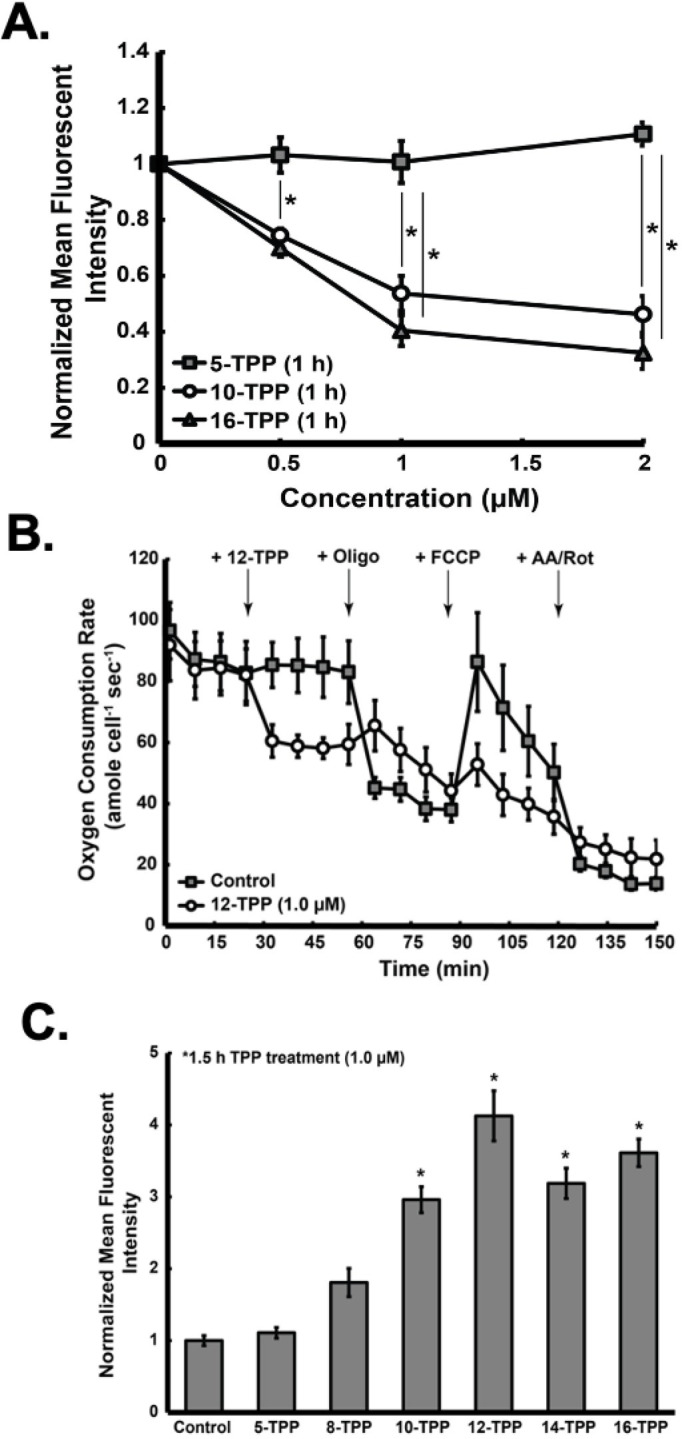
Inclusion of TPP side chains result in decreased melanoma cell mitochondria membrane potential, oxygen consumption, and increased DHE oxidation. (A) A375 melanoma cells were treated with 0.5 μM, 1.0 μM, or 2.0 μM 5-, 10-, or 16-TPP for 1 h and analyzed for mitochondria membrane potential by the JC-1 method (* significant relative to control; p<0.05; n = 2 from 2 separate experiments; N = 4). (B) Oxygen consumption rates were measured using a Seahorse Bioscience XF96 extracellular flux analyzer in A375 melanoma cells treated with or without 1.0 μM 12-TPP (N = 4). At the 25 min mark, 1.0 μM 12-TPP was added to treatment A375 cells followed by the sequential addition of 2.5 μM oligomycin at the 60 min mark, 0.3 μM oligomycin at the 90 min mark, and 5.0 μM rotenone/antimycin A at the 120 min mark. (C) A375 melanoma cells were treated with 1.0 μM 5-, 8-, 10-, 12-,14-, or 16-TPP for 1.5 h and analyzed for DHE oxidation (* significant relative to control; p<0.05; n = 3 from 2 separate experiments; N = 6). The error bars for all data presented in Fig 3 represent the mean ± SEM. Results demonstrate that TPP derivatives preferentially accumulate in mitochondria; decrease mitochondria membrane potential and ATP-linked oxygen consumption; and increase DHE oxidation. Further, there is a structure-activity relationship between TPP side chain length and metabolic disruption; with inclusion of longer side chains resulting in the greatest decreases in mitochondria membrane potential and DHE oxidation.

In order to determine structure-activity relationships of TPP derivatives on mitochondrial membrane potential, A375 melanoma cells were treated with increasing concentrations of TPP-derivatives for 1 h followed by JC1 analysis. Results demonstrate that cells treated with 5-TPP do not exhibit a loss of mitochondria membrane potential relative to untreated controls at all concentrations tested (Fig 3A). In contrast, there was a chain length and concentration dependent decrease in mitochondrial membrane potential in cells treated with 10- or 16-TPP. These results support the hypothesis that TPP-derivatives interact with the mitochondria of melanoma cells and the disruption of this interaction causes decreases in mitochondrial membrane potential. Importantly, since 5-TPP had no effect on mitochondrial membrane potential, these data also support the conclusion that the loss of membrane potential is not due to the cationic TPP moiety, but rather the TPP side chain insertion into the mitochondria membrane. Further, the mitochondria were not completely depolarized when treated with TPP derivatives. These findings support the hypothesis that the decrease in melanoma cell viability and clonogenic survival is not simply attributed to total mitochondria membrane depolarization or disruption, but presumably to a disruption in mitochondrial metabolism.

### TPP decreases oxygen consumption in melanoma cells

In order to determine the effects of the 12-TPP derivative on mitochondrial O_2_ consumption, a mitochondrial stress test was performed [23]. 12-TPP was chosen because previous studies demonstrated that inclusion of side chains longer than 12-TPP did not appreciably enhance the effects on melanoma cell mitochondrial membrane potential. During the experiments, A375 human melanoma cells were treated by sequential addition of: (1) 12-TPP (1.0 μM) or vehicle only; (2) oligomycin to determine mitochondrial ATP-linked oxygen consumption: (3) FCCP to determine mitochondrial reserve capacity; and (4) antimycin A/rotenone to assess for non-mitochondrial sources of oxygen consumption. Results demonstrate that baseline oxygen consumption in A375 human melanoma cells is relatively high (≈90 amol s^-1^ cell^-1^) compared to many types of cancer and non-cancer cells (*e*.*g*, generally less than 50 amol s^-1^ cell^-1 (38)^) (Fig 3B). This finding supports the proposal that melanoma cells have high OCR and ETS activity and provides rationale for a mitochondrial targeted therapy. Immediately upon addition of 12-TPP, OCR decreased significantly compared to cells treated with vehicle only. Since uncouplers of the ETS generally result in an increase in oxygen consumption, this finding suggests that TPP acts as an inhibitor of ETS activity limiting the availability of electrons in complex IV. When oligomycin was added to cells that were not treated with 12-TPP, there was a sharp decrease in oxygen consumption. There was a minimal decrease in oxygen consumption in cells treated with 12-TPP following addition of oligomycin. This also suggests that TPP acts as an inhibitor of the ETS, presumably by decreasing ATP-linked oxygen consumption. Further, the additional slight decrease in OCR could indicate that not enough TPP was added to completely diminish oxygen consumption, and oligomycin eliminated any remaining oxygen consumption capacity in these cells. Addition of FCCP resulted in an increase in oxygen consumption in cells that were not treated with 12-TPP; but no increase in oxygen consumption was observed in cells treated with 12-TPP. This indicates that 12-TPP inhibited the movement of electrons through the ETS, presumably decreasing proton pumping across the mitochondria inner membrane, subsequently decreasing membrane potential and removing the reserve capacity of cells to consume oxygen at complex IV, which is linked to ATP production. Addition of antimycin A/rotenone resulted in a decrease in oxygen consumption in cells; the small remaining OCR for both TPP-treated and untreated cells is attributed to non-mitochondrial sources of oxygen consumption. Because similar non-mitochondrial OCRs are observed in both treated and non-treated cells, the decrease in oxygen consumption observed upon first exposure to 12-TPP is due to disruption of the ETS rather than inhibition of non-mitochondrial sources. The sum of these findings supports the conclusion that TPP specifically disrupts melanoma cell mitochondrial metabolism *via* inhibition of ETS activity that leads to ATP-linked oxygen consumption. Further, this data supports our previous findings that TPP derivatives disrupt specific ETS complex activity (S1 Fig) and increase the ECAR, which suggests that cells are forced into glycolysis as a compensatory mechanism to ETS inhibition (S2 Fig).

### Increasing TPP side-chain length increases DHE oxidation in melanoma cells

Metabolic flux data support the idea that the 12-TPP compound decreases melanoma cell oxygen consumption. However, even in the presence of 12-TPP, the cells consumed oxygen at a high rate relative to most other cancer cells (~60 amol cell^-1^ s^-1^) [38]. A potential consequence of TPP derivatives blocking of the flow of electrons through the ETS is an increase in one-electron reductions of O_2_ to form O_2_^**•**−^ [12,20,21,39,40]. It is believed that in non-malignant cells, as much as 0.1–1% of the ETS electrons can result the formation of O_2_^**•**−^, which then forms O_2_ and H_2_O_2_ *via* superoxide dismutase (SOD) [12,41–43]. By retarding the movement of electrons through the ETS with 12-TPP, it is possible that steady-state levels of O_2_^**•**−^ increase because the residence time of electrons at sites in the ETS that are accessible to O_2_ will increase and this could contribute to the toxicity of 12-TPP in melanoma cells. To evaluate this hypothesis, DHE oxidation assays were performed as a surrogate marker for O_2_^**•**−^ in melanoma cells (Fig 3C). For these experiments, cells were incubated with TPP variants at a concentration of 1.0 μM for 1.5 h and then analyzed for DHE oxidation. Treatment with 10-, 12-, 14-, and 16-TPP caused significant increases in DHE oxidation, relative to controls. These findings support the idea that TPP derivatives of increasing chain lengths lead to increasing inhibition of ETS chains resulting in an increase in one electron reductions of O_2_ to form O_2_^**•**−^ that could contribute to the toxicity seen in Fig 2. In order to determine if O_2_^**•**−^ or H_2_O_2_ levels in melanoma cells contribute to oxidative stress leading to TPP derivative mediated toxicity, A375 melanoma cells were transduced with adenoviral vectors that mediate the overexpression of MnSOD, CuZnSOD and/or Cat (S3A–S3C Fig). Following transfection, cells were treated with 1 μM 12-TPP for 24 hours in the CuZnSOD experiment, and with 1 μM 16-TPP for 24 hours in the MnSOD experiment. Cells were then analyzed for clonogenic survival (S4A and S4B Fig). Results demonstrated that CuZnSOD, MnSOD, and Cat overexpression did not inhibit clonogenic cell killing in melanoma cells treated with TPP derivatives. These results suggest that oxidants other than O_2_^**•**−^ and H_2_O_2_ could be participating in TPP toxicity under these experimental conditions. Since increased ROS can induce lipid peroxidation leading to the formation of lipid hydroperoxides (LOOH), glutathione peroxidase (AdGPx4) was overexpressed using a similar adenoviral strategy to determine if TPP treatment could be inducing cell killing by inducing lipid peroxidation. GPx4 converts LOOH to LOH using GSH as a cofactor [44,45]. For these experiments, A375 cells were transduced with Ad-GPx4 (S3D Fig) and treated with 1 μM 10-, 12-, 14-, or 16-TPP for 24 hours. Cells were then analyzed for clonogenic survival (S4C Fig). In contrast to the results with SOD and Cat there was a trend towards inhibition of TPP derivative toxicity that became significant when melanoma cells overexpressing GPx4 were treated with 16-TPP, relative to 16-TPP alone. These results support the hypothesis that metabolic oxidative stress mediated by lipid hydroperoxides contributes to TPP-mediated cytotoxicity.

### Increasing TPP side-chain length disrupts GSH metabolism in melanoma cells

**Fig 4:**
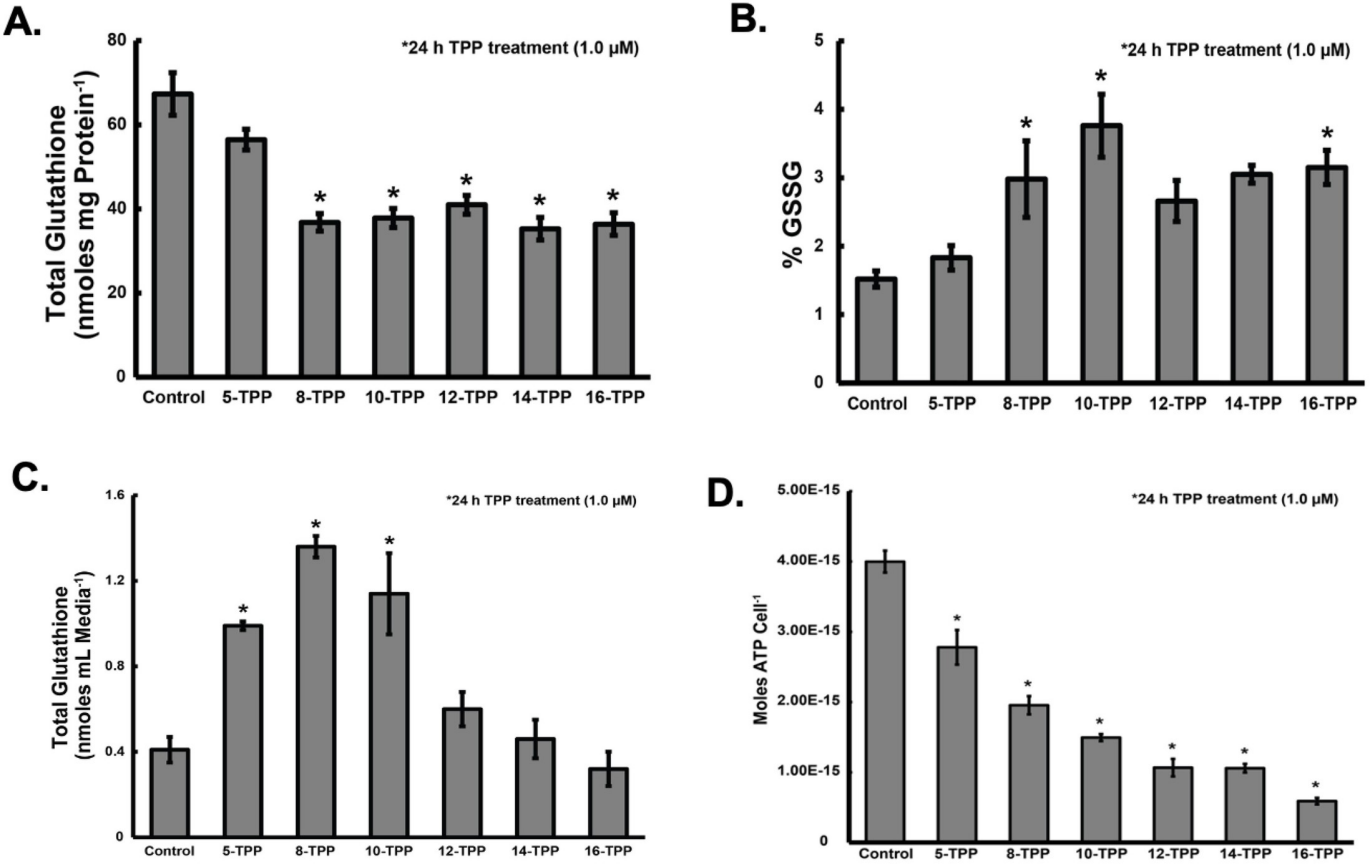
Increasing TPP side-chain length disrupts glutathione metabolism and ATP concentration in melanoma cells. (A) A375 melanoma cells were treated with 1.0 μM 5-, 8-, 10-, 12-,14-, or 16-TPP for 24 h and analyzed for total glutathione (GSH + GSSG) and the percent of total GSH that is GSSG (B) (* significant relative to control; p<0.05; N = 3). (C) A375 melanoma cells were treated with 1.0 μM 5-, 8-, 10-, 12-,14-, or 16-TPP for 24 h. Following treatment, the culture media was collected and analyzed for total GSH (* significant relative to control; p<0.05; N = 3). (D) A375 melanoma cells were treated with 1.0 μM 5-, 8-, 10-, 12-,14-, or 16-TPP for 24 h and analyzed for intracellular ATP concentration with a luminescent-based ATP kit (* significant relative to control; p<0.05; n = 3 from 2 separate experiments; N = 6). The error bars for all data presented in Fig 4 represent the mean ± SEM. Results demonstrate that TPP derivatives decrease total GSH in melanoma cells and increase the percent of total GSH that is GSSG in a chain-length-dependent matter. Results also demonstrate that 5-, 8-, and 10-TPP increase total GSH in the media, whereas 12-, 14-, and 16-TPP do not. ATP measurements demonstrate that TPP compounds decrease intracellular ATP levels; and there are larger decreases in ATP levels as TPP side chain length is increased. Further, 12-, 14-, and 16-TPP resulted in the largest decreases in ATP in melanoma cells. Since GSSG transport is ATP-dependent, this could explain why cells treated with 5-, 8-, and 10-TPP have more total GSH in the tissue culture media compared to cells treated with 12-, 14-, and 16-TPP.

Glutathione functions as a cellular antioxidant that acts as a co-factor for the GPx4 mediated scavenging of LOOH as well as a co-factor for the glutathione transferase (GST) mediated detoxification of toxic lipid aldehydes and other electrophiles derived from the decomposition of LOOH [46–48]. If lipid peroxidation leads to the formation of LOOH and aldehydes that contribute to the biological effects of TPP derivatives, it is expected that GSH metabolism would be disrupted in melanoma cells. A375 human melanoma cells treated with TPP derivatives (1 μM) for 24 h demonstrated that TPP derivatives with side chains of eight carbons or longer caused the depletion of ~50% of cellular total GSH (Fig 4A), relative to untreated cells as well as increase in the % oxidized GSH (GSSG) compared to control cells (Fig 4B). Cells treated with TPP derivatives modified with side chains of eight carbons or longer also demonstrated 2–3 fold increases in the % GSSG, relative to untreated cells. Normally, GSSG is transported out of cells through GSH transporters [49–55]. Studies to evaluate the effect of TPP treatment on total GSH in the media showed treatment with 5-, 8-, and 10-TPP did result in significant increases in extracellular total GSH content (Fig 4C). Interestingly, cells treated with 12-, 14-, or 16-TPP did not have significant increases in total GSH in the media, suggesting that the decreases in intracellular total GSH seen in Fig 4A following treatment with 12-16-TPPs could be the result of increased conjugation of GSH to electrophiles which would cause GSH to be depleted but would not lead to export of GSSG into the media. Collectively, these results demonstrate that TPP derivatives disrupt melanoma cell mitochondrial ETS activity leading to a pro-oxidant intracellular environment causing disruptions in GSH metabolism and there is a structure-activity relationship between decreased GSH and TPP chain length. Furthermore, these results identify a potential target (*e*.*g*., GSH and Trx metabolism) for combination therapies designed to enhance melanoma cells sensitivity to TPP treatment.

### Increasing TPP side-chain length decreases ATP levels in melanoma cells

Since GSH transporters for both GSSG and GS-conjugates to electrophiles are ATP-dependent (49–55), ATP measurements were made to determine how TPP treatment affected melanoma cell ATP levels and to potentially explain the differences in total GSH depletion and transport out of cells. A375 human melanoma cells were treated with TPP derivatives (1.0 μM) for 24 h. Following treatment, cells were analyzed for intracellular ATP concentration using a luminescence-based ATP assay (Fig 4D). Results demonstrate that there is a relationship between TPP side chain length and decreased intracellular ATP concentration, with longer side chains resulting in larger decreases in intracellular ATP concentrations. These findings support the hypothesis that there is a TPP side chain length dependence for optimal melanoma cell mitochondrial disruption, ROS generation, GSH metabolism disruption, and cytotoxicity.

### NAC treatment partially protects melanoma cells from TPP-mediated cytotoxicity

**Fig 5:**
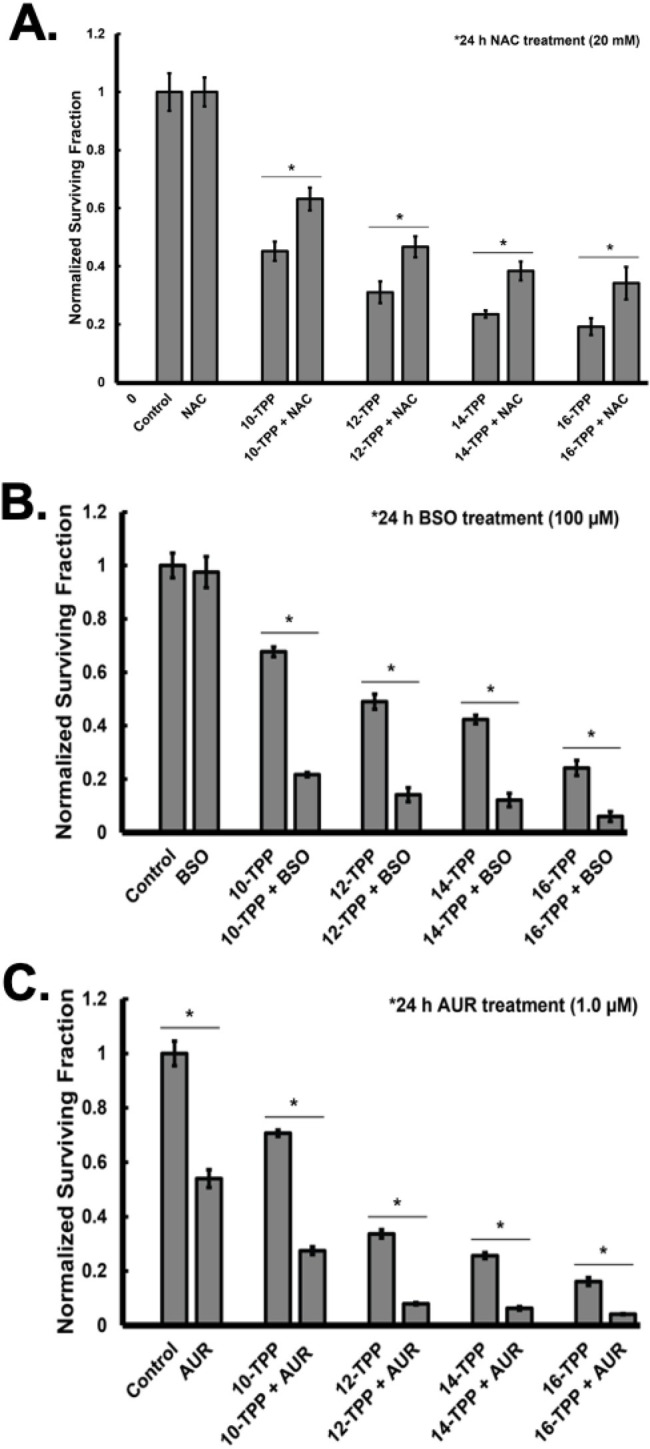
NAC treatment partially protects melanoma cells from TPP mediated cytotoxicity while glutathione synthesis and thioredoxin reductase inhibitors enhance the sensitivity of melanoma cells to TPP treatment. (A) A375 cells were treated with 5–16 TPP alone or in combination with 20 mM N-acetylcysteine (NAC) for 24 h. Following treatment, cells were analyzed for clonogenic survival. (B) A375 cells were treated with 5–16 TPP alone or in combination with 100 μM of the glutathione inhibitor l-buthionine-sulfoximine (BSO) for 24 h. Following treatment, cells were analyzed for clonogenic survival. (C) A375 cells were treated with 5–16 TPP alone or in combination with 1.0 μM of the thioredoxin reductase inhibitor auranofin (AUR) for 24 h. Following treatment, cells were analyzed for clonogenic survival. The error bars for all data presented in Fig 5 (* significant relative to the same TPP drug treatment with NAC, BSO, or AUR; p<0.05; n = 3 from 2 separate experiments; N = 6). Results demonstrate that NAC partially protects melanoma cells from TPP mediated cytotoxicity compared to cells treated with TPP alone. Results also demonstrate BSO and AUR enhance the sensitivity of A375 melanoma cells to TPP treatment compared to TPP alone.

To determine if TPP-induced cytotoxicity functions through ETS inhibition that subsequently results in increased ROS generation and thiol mediated oxidative stress, melanoma cells were treated TPP derivatives in the presence and absence of the non-specific thiol antioxidant NAC, which has been shown to directly scavenge reactive species as well as to provide a source of cysteine necessary for GSH synthesis [56–58]. For these experiments, A375 melanoma cells were treated with 10–16 TPP (1 μM) alone or in combination with 20 mM NAC for 24 h followed by analysis of clonogenic survival (Fig 5A). In all cases NAC was able to significantly protect melanoma cells from TPP induced decreases in clonogenic survival. These results support the hypothesis that TPP compounds disrupt the ETS activity, which increases ROS that leads to thiol-mediated oxidative stress and cytotoxicity.

### Inhibition of GSH synthesis and TrxR activity sensitizes melanoma cells to TPP treatment

To test the working hypotheses that GSH-dependent metabolic detoxification pathways were integrally related to melanoma cell responses to TPP derivatives, clonogenic assays were performed with TPP derivatives (0.5 μM) of 10–16 carbons alone or in combination with 100 μM BSO to inhibit the synthesis of GSH. The results showed that BSO treatment significantly enhanced the cytotoxic effects of all TPP derivatives tested in A375 cells (Fig 5B). These results demonstrate that GSH-dependent pathways are significant determinants of melanoma cell sensitivity to TPP derivatives. In order to test the working hypotheses that Trx-dependent metabolism also represented a significant determinant of melanoma sensitivity to TPP, clonogenic assays were performed using A375 treated with TPP derivatives (0.5 μM) with chain lengths of 10–16 carbons alone or in combination with 1.0 μM AUR, a known inhibitor of TrxR (Fig 5C). Results demonstrate that the addition of AUR was cytotoxic to melanoma cells as well as enhanced cell killing by TPP. Collectively, the results in Fig 5 clearly support the hypothesis that TPP derivative-induced cytotoxicity in melanoma cells is dependent on both GSH and Trx dependent metabolism *via* inducing thiol mediated metabolic oxidative stress.

### The administration route affects TPP tumor accumulation in tumor bearing mice

**Fig 6:**
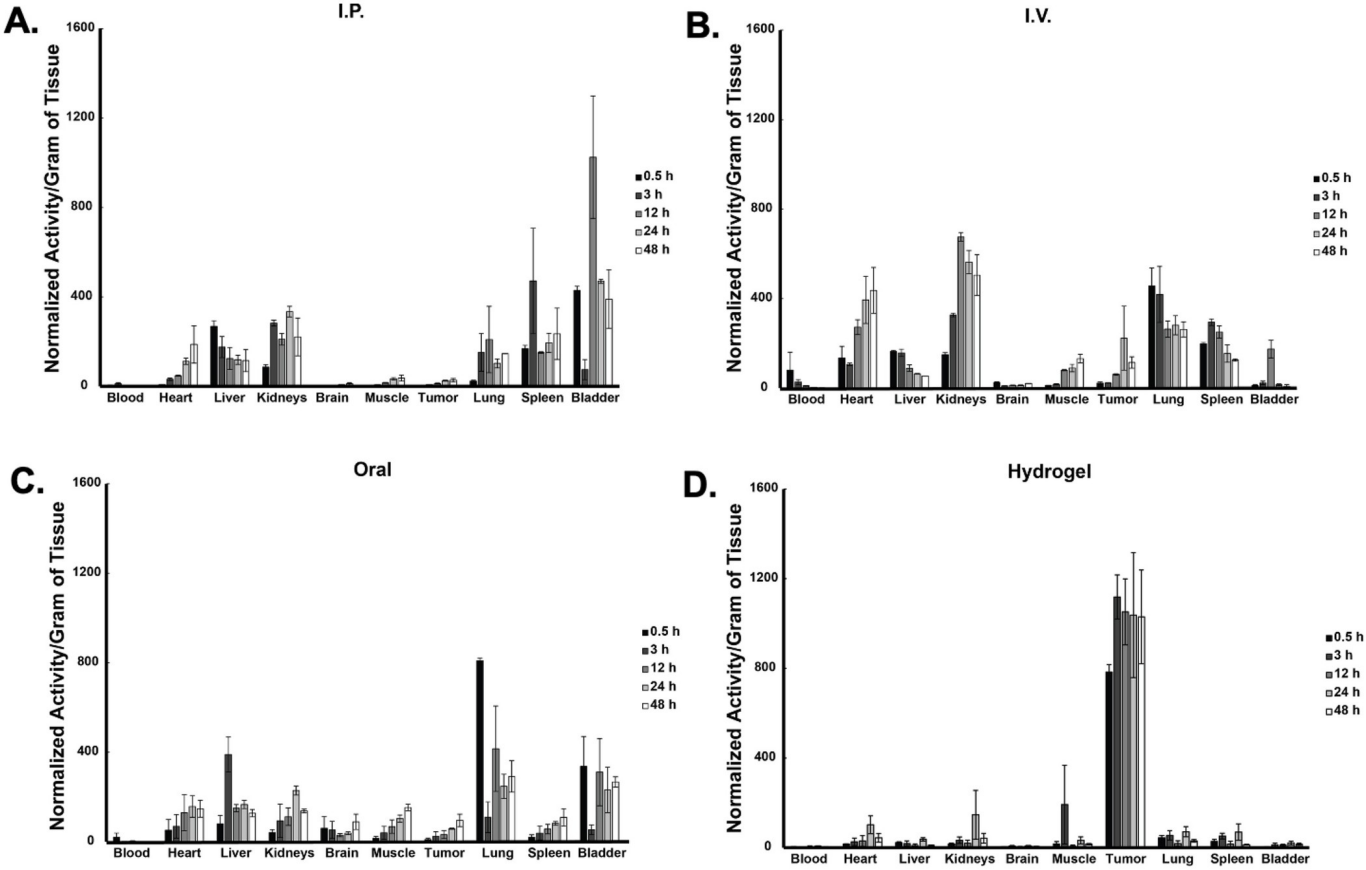
A thermosensitive-hydrogel delivery system results in increased tumor accumulation of TPP compared to traditional routes of drug administration. Mice bearing A375 melanoma tumor xenografts were administered 2.5 μCi of a C-14 labeled 12-TPP via (A) I.P. injection, (B) I.V. injection, (C) oral gavage or (D) a thermosensitive-hydrogel. Following biodistribution times of 0.5 h, 3 h, 12 h, 24 h, or 48 h, animals were sacrificed and tissues harvested. Tissues were ground and counted by liquid scintillation. N = 2 for each route of administration and time point. All data in Fig 6 represent the mean ± SEM. Results demonstrate that a thermosensitive-hydrogel TPP delivery system results in the greatest tumor accumulation of C-14 labeled 12-TPP compared to I.P., I.V. or oral administration routes.

*In vitro* data support the idea that TPP-based compounds have the potential for use as chemotherapeutic agents in melanoma. Despite the potential of TPP compounds as anti-cancer agents *in vitro*, no study to date has demonstrated significant tumor accumulation of TPP compounds *in vivo* or analyzed routes of administration that result in the highest level of tumor accumulation versus normal tissue. To address these issues, a ^14^C-labeled 12-TPP was utilized to determine where 12-TPP accumulates *in vivo* following administration *via* oral gavage, I.V. injection, I.P. injection, or a thermosensitive hydrogel. 12-TPP was chosen because previous *in vitro* studies demonstrated that inclusion of side chains longer than 12-TPP did not significantly increase the cytotoxic effect of TPP on melanoma cell metabolism. Further, inclusion of longer TPP side chains increases the lipophilicity of TPP and would presumably decrease TPP bioavailability. In order to determine the biodistribution of ^14^C-12-TPP following different routes of administration, mice bearing A375 melanoma xenografts were administered 2.5 μCi of ^14^C-12-TPP and sacrificed at different time points. Results demonstrate the ^14^C-12-TPP administered *via* I.P. injection did not result in bioaccumulation in the tumor up to 48 h post-injection (Fig 6A). Administration of ^14^C-12-TPP via I.V. injection resulted in increased tumor accumulation up to 48 h, although it was low compared to other organs analyzed (Fig 6B). Oral administration of ^14^C-12-TPP led to tumor accumulation of TPP that peaked at 48 h, but it was still low compared to other organs analyzed (Fig 6C). In contrast, ^14^C-12-TPP administered via thermosensitive hydrogel, resulted in roughly >10x increase in the amount of detectable ^14^C-12-TPP in tumors as early as 3 h post injection and up to 48 h post injection (Fig 6D). Importantly, little accumulation of ^14^C-12-TPP was evident in all other organs analyzed at all time points. This data strongly suggests that I.P., I.V., and oral administration routes of TPP administration, do not result in favorable accumulation in melanoma tumors compared to off-target organs. This data also strongly supports the potential for use of thermosensitive hydrogels for peritumoral TPP delivery to tumors with little to no off-target bioaccumulation.

### TPP slows melanoma tumor growth when administered *via* hydrogel

**Fig 7:**
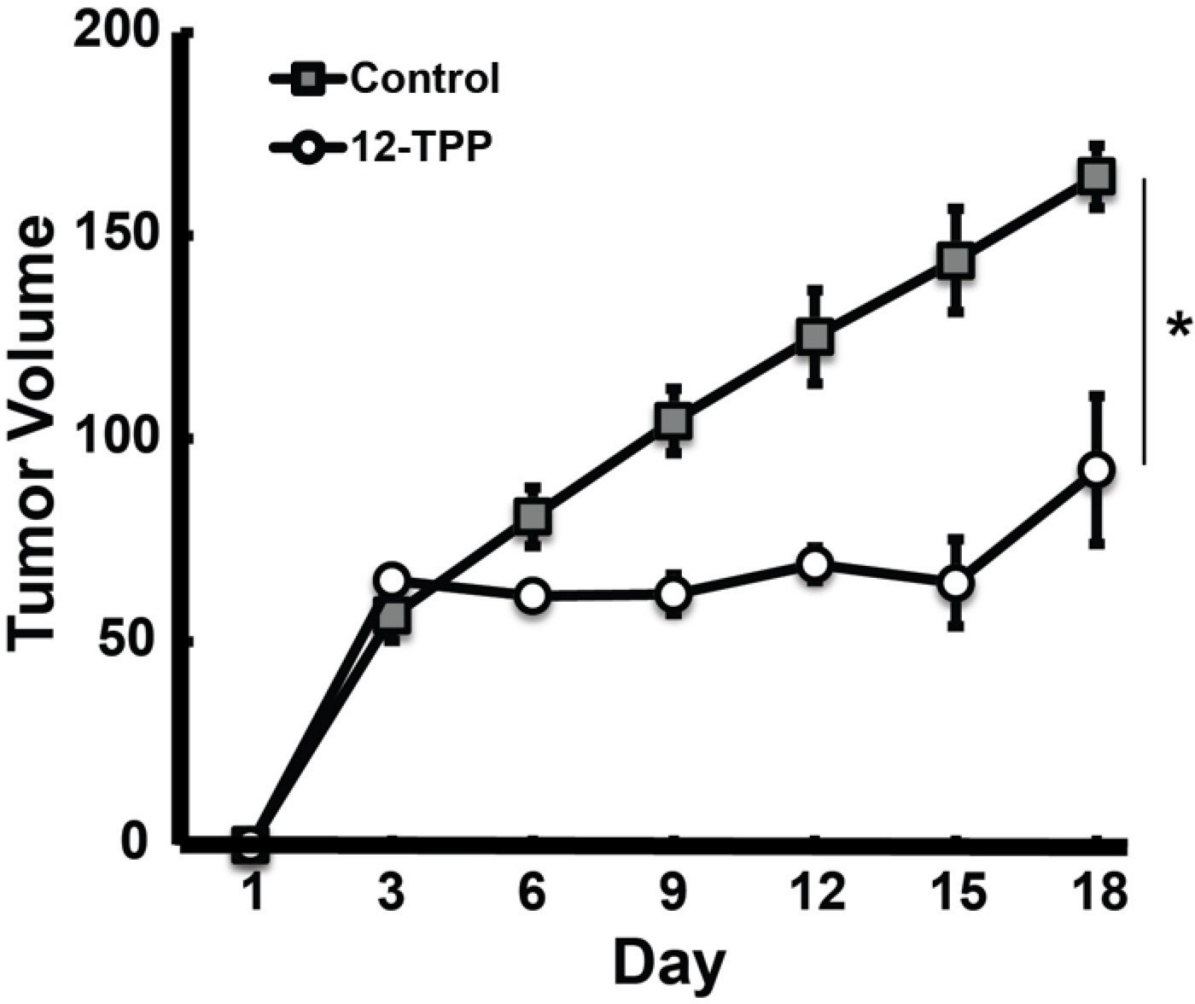
TPP decreases melanoma tumor growth rate when administered via hydrogel. Mice bearing A375 melanoma tumors were administered 10 μM 12-TPP peritumorally via a hydrogel delivery system twice weekly for three weeks. Animal weight and tumor size were recorded twice weekly. Error bars represent standard error of the mean (* significant relative to control; p<0.05; N = 5 mice per group). Results show that 12-TPP administered peritumorally twice weekly for three weeks significantly slowed A375 melanoma tumor growth weight compared to control mice. These results support the continued development of a TPP-hydrogel delivery system for the therapy of metastatic melanoma.

TPP bio-distribution studies demonstrate that a thermosensitive hydrogel delivery system results in 12-TPP-melanoma tumor accumulation as early as 0.5 h post injection and up to 48 h post injection. In order to evaluate the potential for the use of a hydrogel delivery system to administer TPP directly at the tumor site in order to decrease melanoma tumor growth rate, 12-TPP was administered peritumorally via hydrogel to athymic nude mice bearing A375 human melanoma tumors (Fig 7). Mice were treated with hydrogel containing 10 μM 12-TPP or vehicle control twice weekly for three weeks. Tumor size and animal weight was measured at each injection. Animals were sacrificed after three weeks of treatment due to the apparent development of treatment resistance as evidenced by the regrowth of tumors. Results showed that *in vivo* treatment with 12-TPP via hydrogel peritumorally significantly suppressed melanoma tumor growth rate compared to untreated control mice following three weeks of treatment prior to acquiring resistance to treatment. Further, mice maintained body weight and treatment appeared to be well-tolerated. These results suggest that a TPP-hydrogel delivery system has the potential to be an effective therapy adjuvant for the treatment of melanoma.

### TPP treatment does not result in systemic toxicity

*In vivo* efficacy studies demonstrated that 12-TPP administered peritumorally via hydrogel significantly decreases melanoma tumor growth rate compared to untreated mice and appeared to be well tolerated in animals (Fig 7). Biodistribution studies also demonstrated that 12-TPP administered via hydrogel localized in the tumor with minimal accumulation in other organs; whereas orally administered 12-TPP did result in significantly increased 12-TPP accumulation in off-target organs. In order to identify any potential systemic toxicity due to TPP treatment, 100 μM 10-TPP was administered to mice orally (biodistribution studies indicate that this administration route results in TPP accumulation in off-target organs) via drinking water for 16 days. Following treatment, animals were sacrificed and blood was drawn via cardiac puncture for analysis of bone marrow, liver, and kidney toxicity. CBC differential analysis was conducted on blood to determine how 10-TPP treatment affects white blood cell and red blood cell counts (S5A Fig). There were no statistical differences in neutrophil, lymphocyte, and monocyte counts in the treatment versus control mice. There were no significant differences in hemoglobin levels, percent hematocrit, and percent reticulocytes in treatment versus control groups which indicates red blood cells are not affected by 10-TPP treatment. Blood urea nitrogen levels were measured as another indicator of liver toxicity and kidney function (S5B Fig). No significant differences were found in blood urea nitrogen levels in control versus treatment mice, indicating 10-TPP does not cause liver or kidney damage. Finally, for liver toxicity analysis, bilirubin, albumin, alkaline phosphatase, and alanine transaminase levels were measured (S5C Fig). Again, there were no significant differences between treatment and control mice for all four liver toxicity parameters indicating 10-TPP does not induce liver toxicity. Collectively, these results demonstrate that TPP is well-tolerated in mice and does not cause systemic toxicity. Further, since oral administration does lead to off-target TPP accumulation, whereas hydrogel TPP administration does not, the risk of systemic toxicity is even lower for a hydrogel-TPP delivery system.

## Discussion

Melanoma is one of the most aggressive and lethal forms of cancer, and the incidence is increasing worldwide [59–63]. While melanoma identified early can be cured by surgery, metastatic disease can be highly resistant to current therapies [61,64,65]. Prior to 2011, the only FDA-approved melanoma therapies were dacarbazine and high dose interleukin-2, yet both do not improve the median overall survival of melanoma patients [59,61,62,65–67]. Since 2011, much excitement has been generated in the melanoma field, with the development and US Food and Drug Administration approval of novel targeted therapies (*e*.*g*., small molecule BRAF and MEK inhibitors) and immune checkpoint inhibitor therapies (*e*.*g*., Anti-CTLA4 and Anti-PDL-1 antibodies) [61,62,65–67]. Despite the approval of these new therapies for melanoma, patient response and therapy resistance continues to be a barrier to durable responses [59,61,62,64–67]. These observations demonstrate the need to explore novel therapies for melanoma. The current study targets mitochondrial metabolism for the development of therapies for metastatic melanoma that exploit fundamental differences in melanoma mitochondrial metabolism, relative to non-malignant cells. Several studies have suggested that TPP-based drugs can be designed as vehicles that target hyperpolarized mitochondrial membranes of tumor cells. However, a careful study of the structure function relationships of aliphatic side chains has not been accomplished to date [68–71]. The current studies have focused on the chemical constituent side chain as a contributor to the bioactivity of TPP-based drugs that has the potential to act as a determinant of melanoma cell death. The goal of the study was to develop a more detailed understanding of the metabolic interactions of TPP-based drugs in melanoma cell mitochondria, as well as understanding of the pharmacodynamics of TPP-based drugs leading to the highest accumulation of TPP derivative in melanoma tumors.

The results of the current study are consistent with the hypothesis that the molecular structure of the TPP-side chain can be manipulated to enhance cancer cell cytotoxicity [4] with increasing efficacy being related to increasing the length of an aliphatic side chain. These results suggest that modifications of the TPP aliphatic side chain have the potential to induce differential melanoma cell killing and metabolic oxidative stress at lower concentrations, which can contribute to a more detailed understanding of the potential to establish a therapeutic window for TPP-based treatments *in vivo*. In support of this hypothesis, the effect of increased TPP side chain length was found to enhance the effects on melanoma cell mitochondrial membrane potential, DHE oxidation, GSH depletion, increases in %GSSG, and increased sensitivity to agents that disrupt GSH and Trx mediated thiol metabolism. These results are consistent with previous results showing that low micromolar concentrations of TPP cations, such as the TPP-antioxidant MitoQ, can uncouple mitochondria respiration [23,72,73]. However, the current data also support the hypothesis that straight chain aliphatic TPP derivatives at 1.0 μM concentration) also inhibit melanoma cell ATP-linked oxygen consumption by slowing the movement of electrons down ETSs consistent with previous reports that longer TPP side chains impart greater effects on oxygen consumption rate [4,23,25]. Interestingly, emerging evidence demonstrates that melanoma cells have sustained or heightened oxidative phosphorylation activity that contributes to metabolic stress and the malignant phenotype [74–76]. A significant consequence of heightened oxidative phosphorylation in cancer cells (relative to non-malignant cells) is an increase in ETS generated ROS due to inefficient electron transfer [41–43]. The current results show that TPP disrupts ATP-linked oxygen consumption; and this disruption increases in one electron reductions of O_2_ to form O_2_^**•**−^, as evidenced by increased DHE oxidation in a side chain–length-dependent manner. Interestingly, overexpression of O_2_^**•**−^- and H_2_O_2_-scavenging enzymes did not protect melanoma cells from TPP toxicity. In contrast, GPx4 over expression and NAC did protect melanoma cells from TPP-mediated cytotoxicity, suggesting a significant role for lipid peroxidation with the formation of lipid hydroperoxides and aldehydes in TPP toxicity, leading to thiol mediated oxidative stress. Consistent with this hypothesis, TPP derivatives were found to deplete intracellular total GSH leading to an increase in % GSSG. Further, the media containing cells treated with TPP compounds also showed elevated levels of extracellular total GSH. When GSH is oxidized to GSSG or conjugated to an electrophile by the actions of glutathione peroxidase or glutathione transferase enzymes, it is transported out of cells. However, in the present study, elevated total GSH in media was only observed in cells treated with 5-, 8-, and 10-TPP and not in cells treated with 12-, 14-, or 16-TPP. One possible explanation for these results is that GSSG and GS-conjugate transport is carried out by transporters that are ATP dependent and the longer chain TPP derivatives cause a more severe depletion of ATP. Clonogenic survival studies demonstrated that NAC is able to inhibit TPP induced cytotoxicity, which suggests that TPP causes cytotoxicity due to ETS disruption, subsequent ROS generation, and disruption of thiol metabolism leading to thiol mediated oxidative stress. In support of this hypothesis, inhibition of GSH synthesis with BSO or inhibition of TRxR with AUR was found to further enhance the sensitivity of melanoma cells to TPP treatment. Collectively, these results support the conclusion that TPP derivatives with greater than 10-carbon aliphatic side chains disrupt ETS mediated oxygen consumption, increase ROS levels, and caused disruptions of GSH metabolism leading to thiol mediated oxidative stress and cell killing in melanoma cells. When the route of administration that results in the highest TPP derivative tumor accumulation was considered, the extent of uptake of TPP in cancer cells and cancer cell mitochondria was thought to be dependent upon the hydrophobicity of TPP drugs. Furthermore, the anchoring of the TPP derivative side chain into the mitochondria membrane and effect on mitochondria and cellular metabolism was also thought to be dependent on the lipophilicity of TPP [23]. It should also be noted that TPP concentrations utilized *in vitro* in many studies have not yet been determined to be reasonably achievable following metabolism and clearance in animals or humans [23]. Our results showed that systemic administration (*e*.*g*., I.P., I.V., oral) of TPP derivatives with aliphatic side chains (^14^C-12-TPP) lead to greater TPP accumulation in off-target tissues with very modest tumor drug accumulation. In order to identify potential normal tissue toxicity associated with systemic TPP treatment, mice were administered 100 μM 10-TPP *via* drinking water with the expectation of significant off-target TPP accumulation based on the biodistribution studies. In this experiment there was no apparent liver, kidney, or bone marrow toxicity after 17 days of treatment demonstrating the ability of the animals to tolerate the off-target effects of TPP derivatives. Based on this knowledge, we also explored a new and potentially exciting alternative approach to drug administration using a thermosensitive-hydrogel that exists in a water-like state at low temperatures and forms a gel at physiological temperature, allowing for the controlled release of a lipophilic drug at a desired site. While many thermosensitive hydrogels have been explored for the intra- or peri-tumoral delivery of chemotherapeutic drugs, surgical resection followed chemotherapy or radiation remains the primary method to remove primary and metastatic lesions [77–79]. A major challenge to clinical translation of thermosensitive hydrogels is the need for improved chemical formulations to improve hydrogel gelling, drug loading and release, and hydrogel degradation properties [77]. Hydrogel drug combinations must also be hand injectable and visible upon injection [77]. Fortunately, ongoing efforts continually improve hydrogel formulations for intra- or peri-tumoral delivery of chemotherapeutic drugs. Further, advances in CT and ultrasound imaging technology have the potential to make image-guided administration of thermosensitive hydrogels more precise and practical [77,78]. Biodistribution studies demonstrated that ^14^C-12-TPP administered via hydrogel peritumorally was detectable at significant levels for at least 48 h post injection in melanoma tumors, with minimal accumulation in off target organs. This route of administration was also found to cause significant tumor growth delay for three weeks in A375 melanoma tumors *in vivo*. These findings suggest that hydrogel-based TPP derivative delivery systems could be an alternative administration route that results in excellent and effective drug delivery to the tumor site. Importantly, the hydrogel delivery systems are capable of delivering a payload directly at the tumor site and therefore also limit systemic toxicity associated with chemotherapy. A hydrogel delivery system could also be loaded with multiple anti-cancer agents in order to target multiple cellular components and a way to circumvent cancer resistance [80]. Hydrogel loaded with TPP could also be applied to tumor margins following surgical resection and potentially decrease the likelihood of tumor recurrence. Lastly, a hydrogel-based TPP delivery system could potentially be injected at superficial tumor sites and minimize scarring often associated with surgical resection. Overall, our results highlight the potential utility of a TPP derivative hydrogel delivery system for cancer therapy.

## Supporting information

S1 Fig TPP derivatives inhibit mitochondria ETS activity. The activity of ETS complexes I-IV was measured in enriched mitochondria treated with 10 μM 10-TPP. Error bars represent the standard error of the mean (N = 2). These results support that 10-TPP inhibits ETS complexes I and III activity relative to untreated controls and supports the hypothesis that TPP compounds disrupt mitochondria oxidative metabolism.
(TIF)

S2 Fig TPP derivatives increase melanoma cell glycolytic activity. A375 melanoma cells were plated in XF96 plates and incubated for 48 h. Extracellular acidification rate (ECAR) measurements were made using a Seahorse Bioscience XF96 extracellular flux analyzer for 150 min. (1) 12-TPP was injected at the 20 min mark followed by the sequential addition of (2) oligomycin, (3) FCCP, and (3) antimycin A and rotenone. Results show that 12-TPP treatment immediately increases the ECAR in A375 melanoma cells. Error bars represent the standard error of the mean (N = 4). These results support the hypothesis that TPP interferes with mitochondria oxidative metabolism via ATP-linked oxygen consumption, which forces cells into glycolysis as a compensatory mechanism to meet metabolic requirements.
(TIF)

S3 Fig Melanoma cells transfected with a CuZnSOD, MnSOD, catalase, and GPx4 adenovirus exhibit increased enzyme activity. A375 cells were plated in 60 mm tissue culture dishes and incubated for 48 h. Cells were then transfected with Ad-CuZnSOD, Ad-MnSOD, Ad-Cat, or Ad-GPx4 for 24 h in serum free media. The adenovirus was then removed and full media replaced for 24 h. Cells were then analyzed for (A) CuZnSOD and (B) MnSOD activity by measuring the rate of NBT reduction by O_2_^•−^ spectrophotometrically (N = 1). Cells were analyzed for (C) Cat activity by measuring the rate of H_2_O_2_ decay spectrophotometrically (N = 1). Cells were analyzed for (D) GPx4 activity by measuring the rate of NADPH oxidation by GSSG due to the oxidation of glutathione by lipid hydroperoxides and GPx4 spectrophotometrically. (* significant relative to control, p < 0.05, N = 3). Results demonstrate that A375 melanoma cells can be successfully transfected with SOD, Cat, and GPx4 adenovirus and exhibit high enzymatic activity following transfection.
(TIF)

S4 Fig CuZnSOD, MnSOD, Cat, and GPx4 overexpression do not protect melanoma cells from TPP-mediated cytotoxicity. A375 cells were plated in 60 mm tissue culture dishes and incubated for 48 h. Cells were then transfected with adenovirus (Ad-CuZnSOD, Ad-MnSOD, Ad-Cat, and Ad-GPx4) for 24 h in serum free media. The adenovirus was then removed and full media replaced for 24 h. Cells were then treated with 1 μM 12-TPP for 24 h. Following drug treatment, cells were plated for a clonogenic survival assay. (A) Results demonstrate that CuZnSOD and Cat do not protect melanoma cells from TPP-mediated cytotoxicity (N = 3). (B) Results also show that MnSOD and Cat do not protect melanoma cells from TPP mediated cytotoxicity. (n = 3 from 3 separate experiments; N = 6). (C) Further, GPx4 does not protect melanoma cells from TPP-mediated cytotoxicity (* significant relative to the same TPP drug treatment with Ad-GPx4, p < 0.05, n = 3 from 2 separate experiments, N = 6). Error bars for all data presented in S2 Fig represent the standard error of the mean. Collectively, these results suggest that O_2_^•-^ and H_2_O_2_ are likely not the specific ROS responsible for the DHE oxidation observed in Fig 3C.
(TIF)

S5 Fig TPP treatment does not cause bone marrow, liver, or kidney toxicity following oral administration for 17 days. Mice were administered 100 μM 10-TPP via drinking water for 17 days (this administration route results in TPP accumulation in organs (Fig 6D). Following treatment, animals were sacrificed and blood was drawn via cardiac puncture. White blood cell (WBC) counts and red blood cell (RBC) counts were determined by CBC analysis (A). (B) Blood urea nitrogen levels (BUN; an indicator of liver and kidney function) and markers of liver function (C) (Bili, albumin, ALP, ALT) were also measured. Results indicate no significant differences in WBC parameters (HGB, HCT, RET) and RBC parameters (HGB, HCT, RET) between treatment and control mice. These results indicate that TPP treatment does not affect the bone marrow. There were no differences in BUN levels between treatment and control mice. These results indicate that TPP treatment does not affect liver or kidney function. Finally, there were no differences in bili, albumin, ALP, and ALT levels between treatment and control mice. These results indicate that TPP treatment does not cause liver toxicity. Collectively, since biodistribution studies demonstrate that oral administration results in off-target TPP accumulation in normal organs whereas TPP administered via hydrogel does not, and oral administration does not cause liver, kidney, or bone marrow damage, TPP administered via hydrogel might not cause toxicity in not malignant tissues.
(TIF)

S1 File (PDF)
