# Supplementary figures and images for: Triphenylphosphonium derivatives disrupt metabolism and inhibit melanoma growth in vivo when delivered via a thermosensitive hydrogel
Source: PLoS One. 2020 Dec 30;15(12):e0244540. doi: 10.1371/journal.pone.0244540 (PMC7773266; doi:10.1371/journal.pone.0244540)

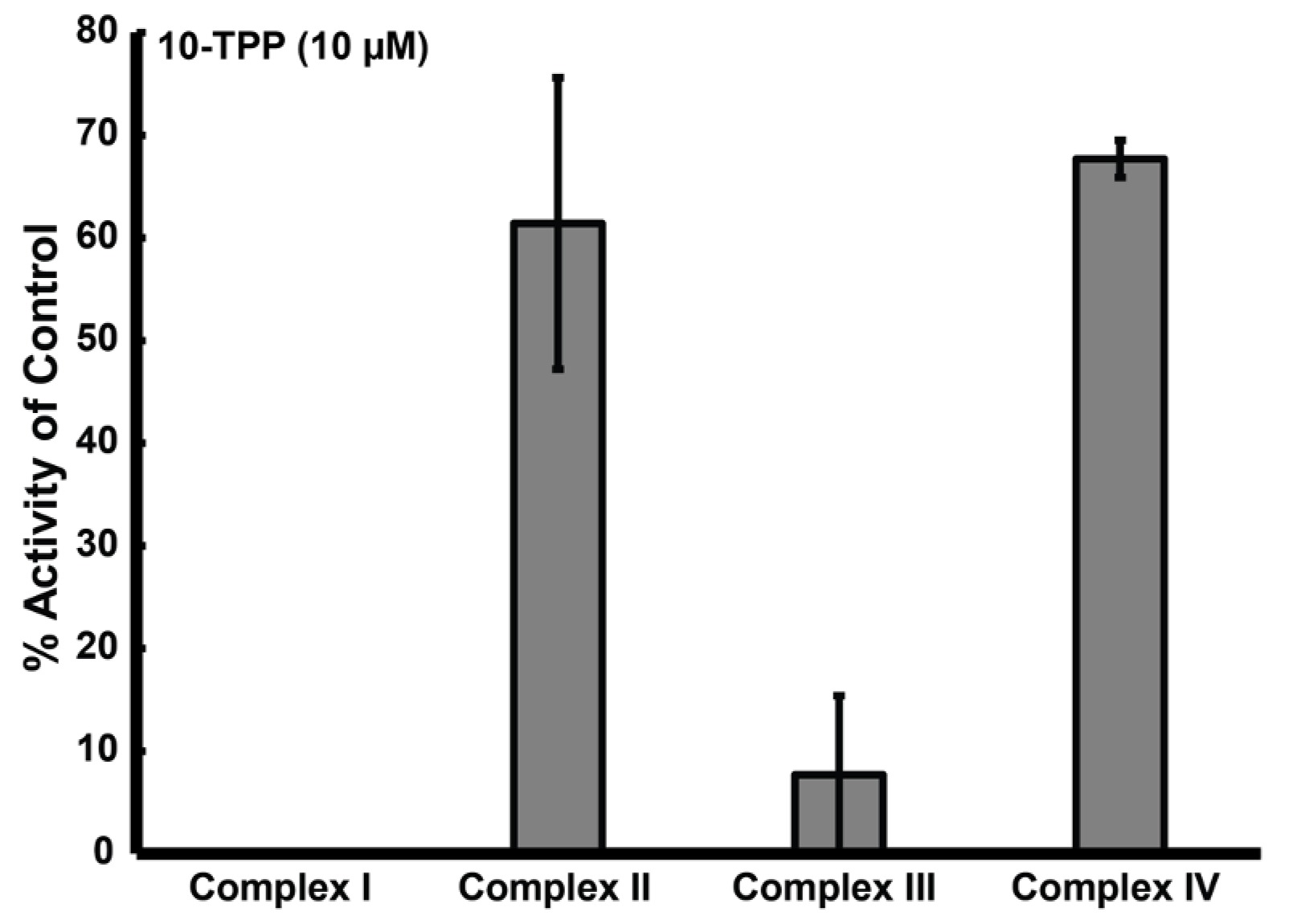

Supplement: S1 Fig — The activity of ETS complexes I-IV was measured in enriched mitochondria treated with 10 μM 10-TPP. Error bars represent the standard error of the mean (N = 2). These results support that 10-TPP inhibits ETS complexes I and III activity relative to untreated controls and supports the hypothesis that TPP compounds disrupt mitochondria oxidative metabolism. (TIF) [file pone.0244540.s001.tif]

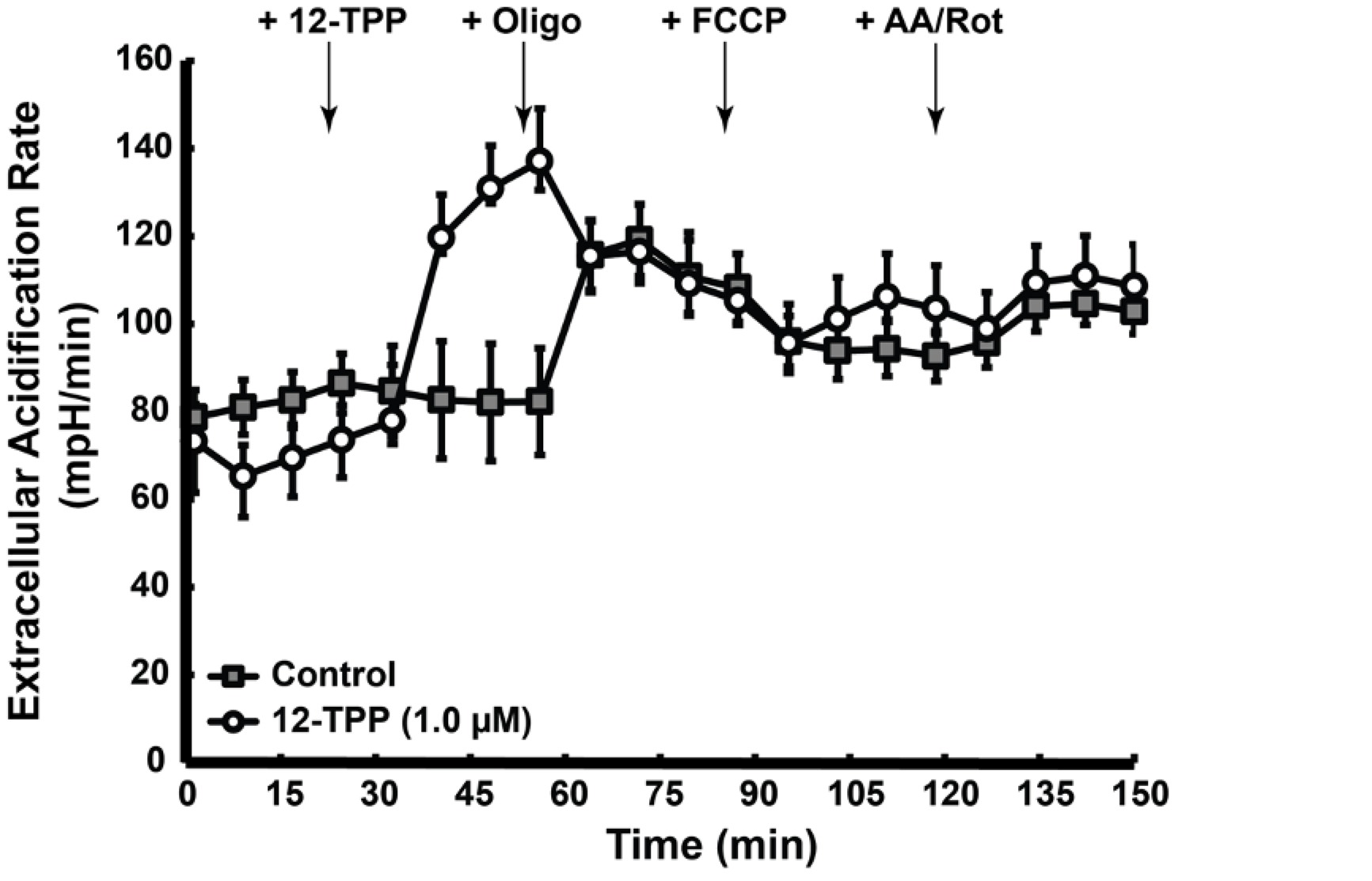

Supplement: S2 Fig — A375 melanoma cells were plated in XF96 plates and incubated for 48 h. Extracellular acidification rate (ECAR) measurements were made using a Seahorse Bioscience XF96 extracellular flux analyzer for 150 min. (1) 12-TPP was injected at the 20 min mark followed by the sequential addition of (2) oligomycin, (3) FCCP, and (3) antimycin A and rotenone. Results show that 12-TPP treatment immediately increases the ECAR in A375 melanoma cells. Error bars represent the standard error of the mean (N = 4). These results support the hypothesis that TPP interferes with mitochondria oxidative metabolism via ATP-linked oxygen consumption, which forces cells into glycolysis as a compensatory mechanism to meet metabolic requirements. (TIF) [file pone.0244540.s002.tif]

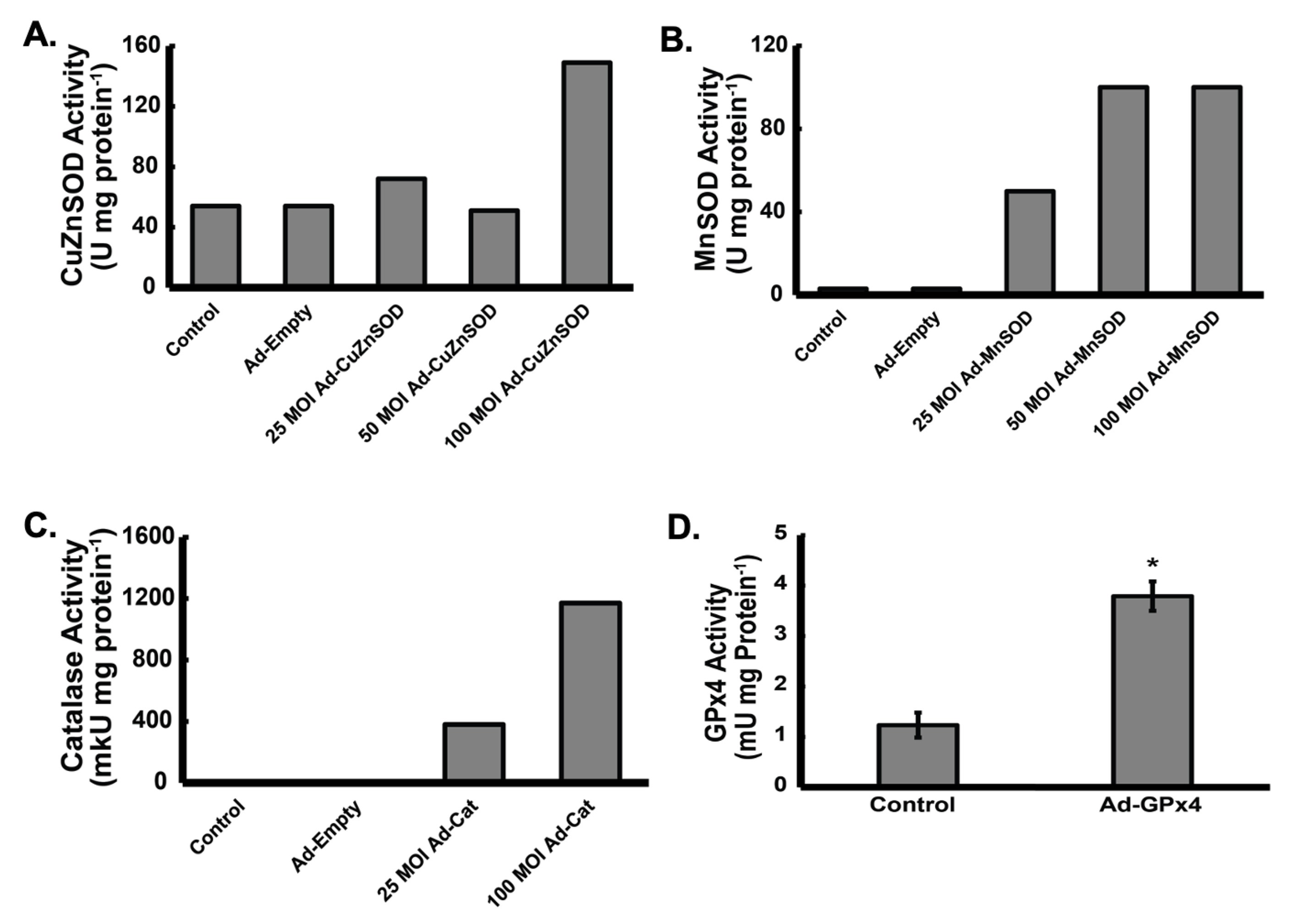

Supplement: S3 Fig — A375 cells were plated in 60 mm tissue culture dishes and incubated for 48 h. Cells were then transfected with Ad-CuZnSOD, Ad-MnSOD, Ad-Cat, or Ad-GPx4 for 24 h in serum free media. The adenovirus was then removed and full media replaced for 24 h. Cells were then analyzed for (A) CuZnSOD and (B) MnSOD activity by measuring the rate of NBT reduction by O2•− spectrophotometrically (N = 1). Cells were analyzed for (C) Cat activity by measuring the rate of H2O2 decay spectrophotometrically (N = 1). Cells were analyzed for (D) GPx4 activity by measuring the rate of NADPH oxidation by GSSG due to the oxidation of glutathione by lipid hydroperoxides and GPx4 spectrophotometrically. (* significant relative to control, p < 0.05, N = 3). Results demonstrate that A375 melanoma cells can be successfully transfected with SOD, Cat, and GPx4 adenovirus and exhibit high enzymatic activity following transfection. (TIF) [file pone.0244540.s003.tif]

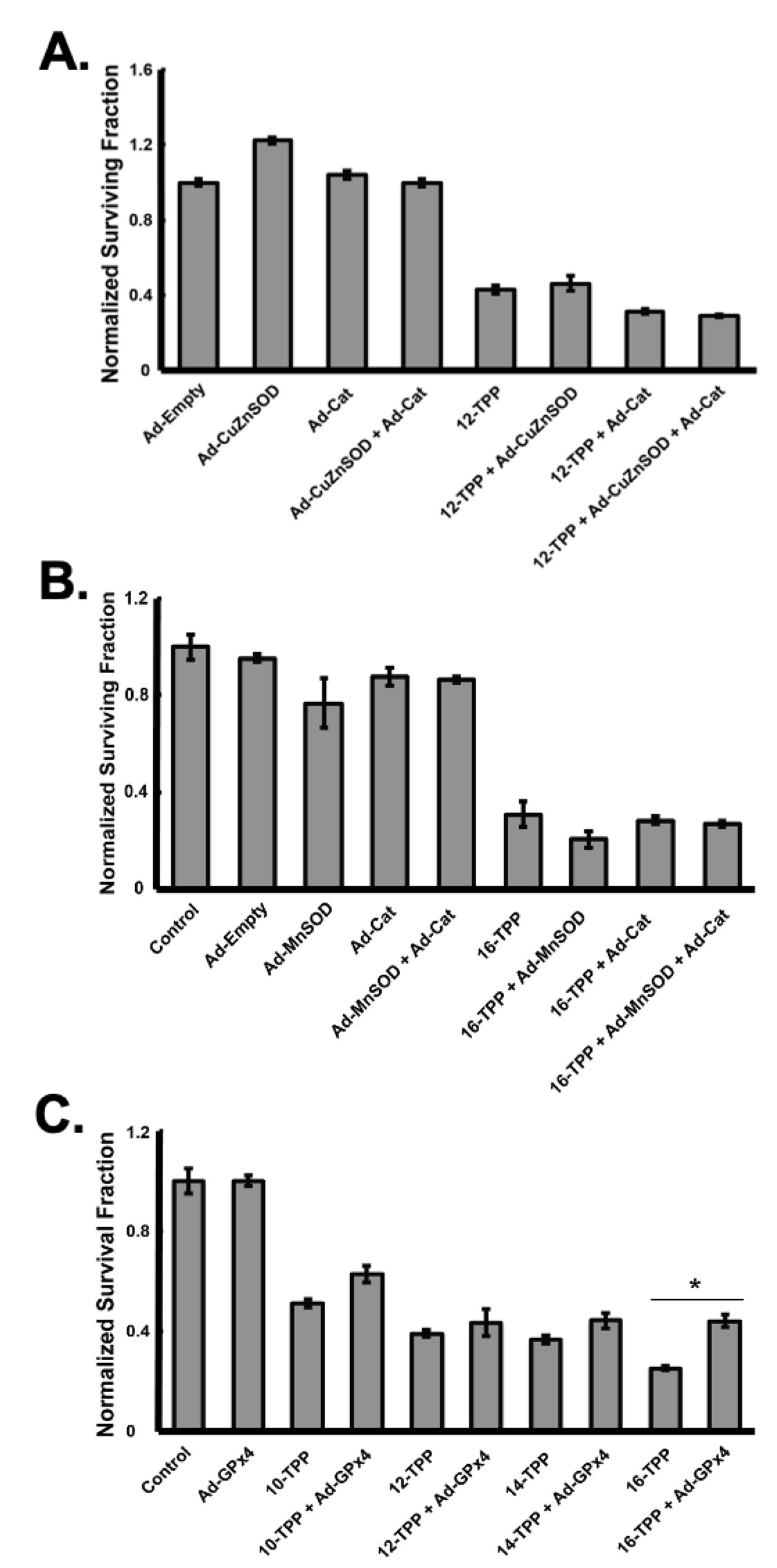

Supplement: S4 Fig — A375 cells were plated in 60 mm tissue culture dishes and incubated for 48 h. Cells were then transfected with adenovirus (Ad-CuZnSOD, Ad-MnSOD, Ad-Cat, and Ad-GPx4) for 24 h in serum free media. The adenovirus was then removed and full media replaced for 24 h. Cells were then treated with 1 μM 12-TPP for 24 h. Following drug treatment, cells were plated for a clonogenic survival assay. (A) Results demonstrate that CuZnSOD and Cat do not protect melanoma cells from TPP-mediated cytotoxicity (N = 3). (B) Results also show that MnSOD and Cat do not protect melanoma cells from TPP mediated cytotoxicity. (n = 3 from 3 separate experiments; N = 6). (C) Further, GPx4 does not protect melanoma cells from TPP-mediated cytotoxicity (* significant relative to the same TPP drug treatment with Ad-GPx4, p < 0.05, n = 3 from 2 separate experiments, N = 6). Error bars for all data presented in S2 Fig represent the standard error of the mean. Collectively, these results suggest that O2•- and H2O2 are likely not the specific ROS responsible for the DHE oxidation observed in Fig 3C. (TIF) [file pone.0244540.s004.tif]

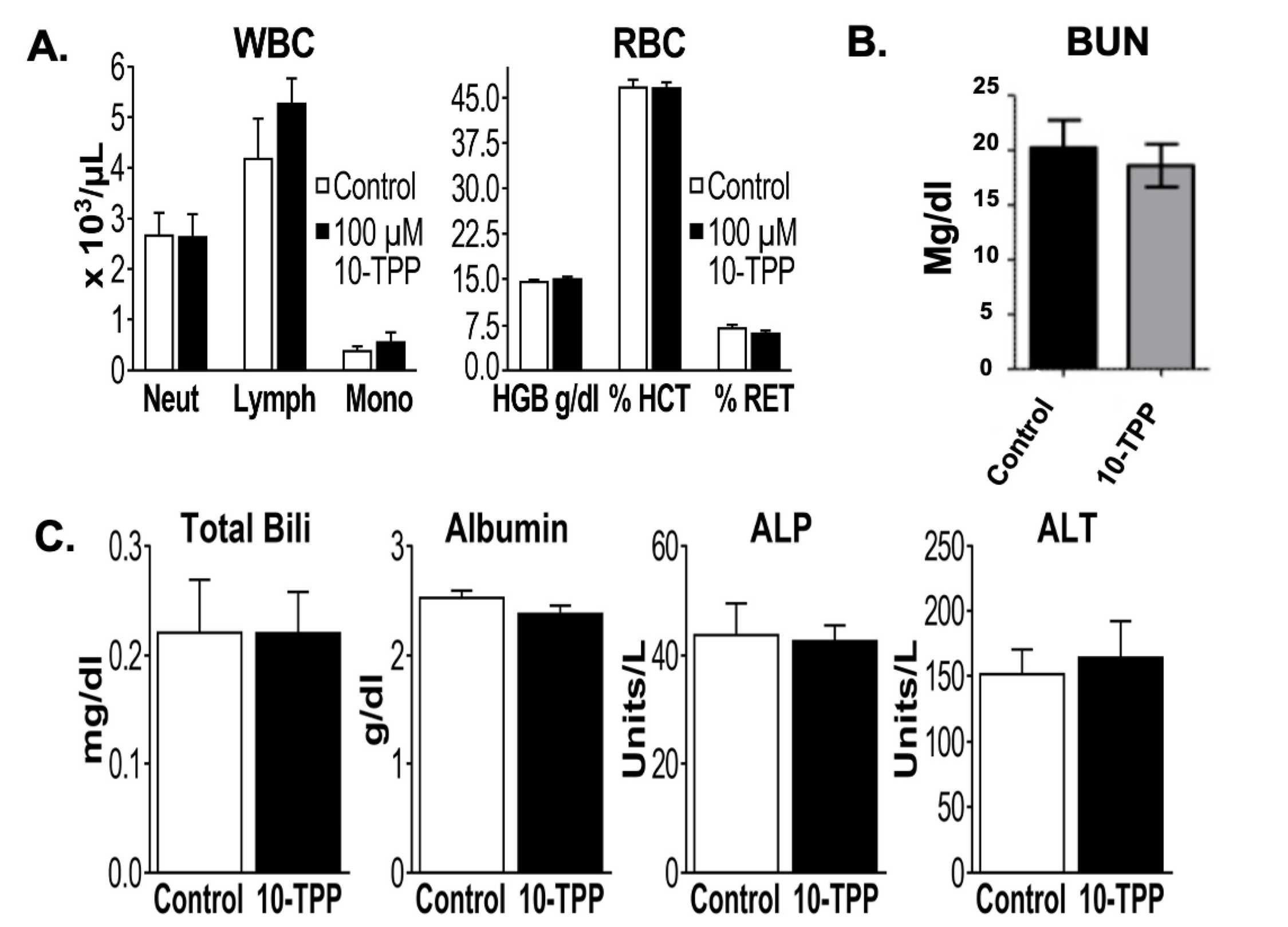

Supplement: S5 Fig — Mice were administered 100 μM 10-TPP via drinking water for 17 days (this administration route results in TPP accumulation in organs (Fig 6D). Following treatment, animals were sacrificed and blood was drawn via cardiac puncture. White blood cell (WBC) counts and red blood cell (RBC) counts were determined by CBC analysis (A). (B) Blood urea nitrogen levels (BUN; an indicator of liver and kidney function) and markers of liver function (C) (Bili, albumin, ALP, ALT) were also measured. Results indicate no significant differences in WBC parameters (HGB, HCT, RET) and RBC parameters (HGB, HCT, RET) between treatment and control mice. These results indicate that TPP treatment does not affect the bone marrow. There were no differences in BUN levels between treatment and control mice. These results indicate that TPP treatment does not affect liver or kidney function. Finally, there were no differences in bili, albumin, ALP, and ALT levels between treatment and control mice. These results indicate that TPP treatment does not cause liver toxicity. Collectively, since biodistribution studies demonstrate that oral administration results in off-target TPP accumulation in normal organs whereas TPP administered via hydrogel does not, and oral administration does not cause liver, kidney, or bone marrow damage, TPP administered via hydrogel might not cause toxicity in not malignant tissues. (TIF) [file pone.0244540.s005.tif]
